# Pathogenicity and virulence of Crimean-Congo hemorrhagic fever virus: From enzootic maintenance to severe human disease

**DOI:** 10.1080/21505594.2026.2709250

**Published:** 2026-08-05

**Authors:** Virginia Aida-Ficken, Teresa E. Sorvillo, Elif Karaaslan, Katherine A. Davies, Éric Bergeron, Stephen R. Welch, Jessica R. Spengler

**Affiliations:** a Foreign Animal Disease Diagnostic Laboratory, National Veterinary Services Laboratories, National Bio and Agro-Defense Facility, United States Department of Agriculture, Manhattan, KS, USA; b Viral Special Pathogens Branch, Division of High-Consequence Pathogens and Pathology, Centers for Disease Control and Prevention, Atlanta, GA, USA; c Infectious Disease Department, CDC Foundation, Atlanta, GA, USA; d Department of Infectious Diseases, College of Veterinary Medicine, University of Georgia, Athens, GA, USA

**Keywords:** Crimean-Congo hemorrhagic fever virus, virulence, pathogenesis, tick-borne disease, host–pathogen interactions, immune evasion

## Abstract

Crimean-Congo hemorrhagic fever (CCHF) is a severe tick-borne disease caused by Crimean-Congo hemorrhagic fever virus (CCHFV), an enveloped, negative-sense, tri-segmented RNA virus in the genus *Orthonairovirus*. The virus is endemic across Africa, the Middle East, Asia, and Europe, shaped by the ecology of ixodid ticks and their wildlife hosts. While infection in most vertebrates is asymptomatic, human disease ranges from mild illness to fulminant hemorrhagic fever with vascular leak, multiorgan failure, coagulopathy, and shock, with case-fatality rates up to 30% and no licensed countermeasures. This review synthesizes current understanding of CCHFV pathogenicity and virulence across its enzootic cycle. We highlight host and viral factors driving infection in ticks, intermediate hosts, and humans, including entry, immune evasion, and mechanisms underlying severe disease. We also identify key knowledge gaps and research priorities to advance understanding of CCHFV pathogenesis and inform strategies to mitigate severe disease.

## Background and scope

Crimean-Congo hemorrhagic fever (CCHF) is a tick-borne illness caused by infection with the CCHF virus (CCHFV; *Orthonairovirus haemorrhagiae*), with clinical manifestations ranging from mild disease to severe, potentially fatal illness [1]. It is estimated that 10,000–15,000 cases of CCHF occur annually with 88% of cases believed to be subclinical or unrecognized [2–4]. CCHF is the most widely distributed tick-borne viral disease in humans [5,6]. Disease was first recognized in the 1940s in the Crimean region of the former Soviet Union and subsequently isolated in the 1950s in the Belgian Congo (presently DRC). The virus has since been found to be endemic to Africa, the Middle East, Asia, and Europe [7–10]. This transcontinental distribution reflects the broad and expanding geographic range of ixodid tick reservoirs and the wide distribution of their wild vertebrate hosts that serve as amplifying hosts for CCHFV [11–13].

CCHFV is an enveloped negative-sense, tri-segmented, single-stranded RNA virus belonging to the genus *Orthonairovirus* in the family *Nairoviridae* of the order *Hareavirales* [14,15]. The virus is maintained in nature through vertical and horizontal transmission in ticks and can be transmitted from these infected ticks to their avian or mammalian hosts during blood meals [11]. Besides transmission through tick bites, infection in humans can also occur through contact with patients during the acute phase of illness [16,17] or with blood or tissues of viremic animals [16,18–20]. Notably, cases of possible sexual transmission have also been reported [21,22]. Humans can demonstrate a spectrum of disease ranging from mild clinical symptoms to nonspecific febrile syndrome characterized by vascular leak and hemorrhage, multiorgan failure, disseminated intravascular coagulation (DIC), and shock [23]. The case fatality rate ranges from 5% to greater than 30% [24]. To date there are no licensed medical countermeasures. Treatment is primarily supportive in conjunction with the off-label use of Ribavirin with uncertain efficacy [25].

This review synthesizes the current understanding of CCHFV pathogenesis and virulence, highlighting insights gained from both clinical research and experimental models. We first contextualize disease processes within the broader enzootic cycle involving the tick vector and reservoir, vertebrate hosts, and humans. We then examine the identified and proposed molecular mechanisms associated with disease in humans. Finally, we highlight key knowledge gaps and research priorities that are critical to advancing our understanding of CCHFV pathogenesis.

## Pathogenicity and virulence in ticks and livestock

### Ticks

Ticks are hematophagous arthropods divided into two major families: *Ixodidae* (ixodid, hard ticks) and *Argasidae* (argasid, soft ticks) [26]. Ixodid ticks have been identified as both vectors and reservoirs of CCHFV, capable of maintaining the virus throughout their developmental cycle and facilitating transmission to other ticks and vertebrate hosts. In contrast, available experimental evidence does not support argasid ticks as either vectors or reservoirs as they do not appear to sustain CCHFV infection nor transmit the virus to other hosts [27,28]. Among ixodid ticks, *Hyalomma* species are considered the principal reservoirs and vectors of CCHFV, although other genera, including *Dermacentor* and *Rhipicephalus* spp., have also been implicated in the virus life cycle [29].

**Figure 1.**
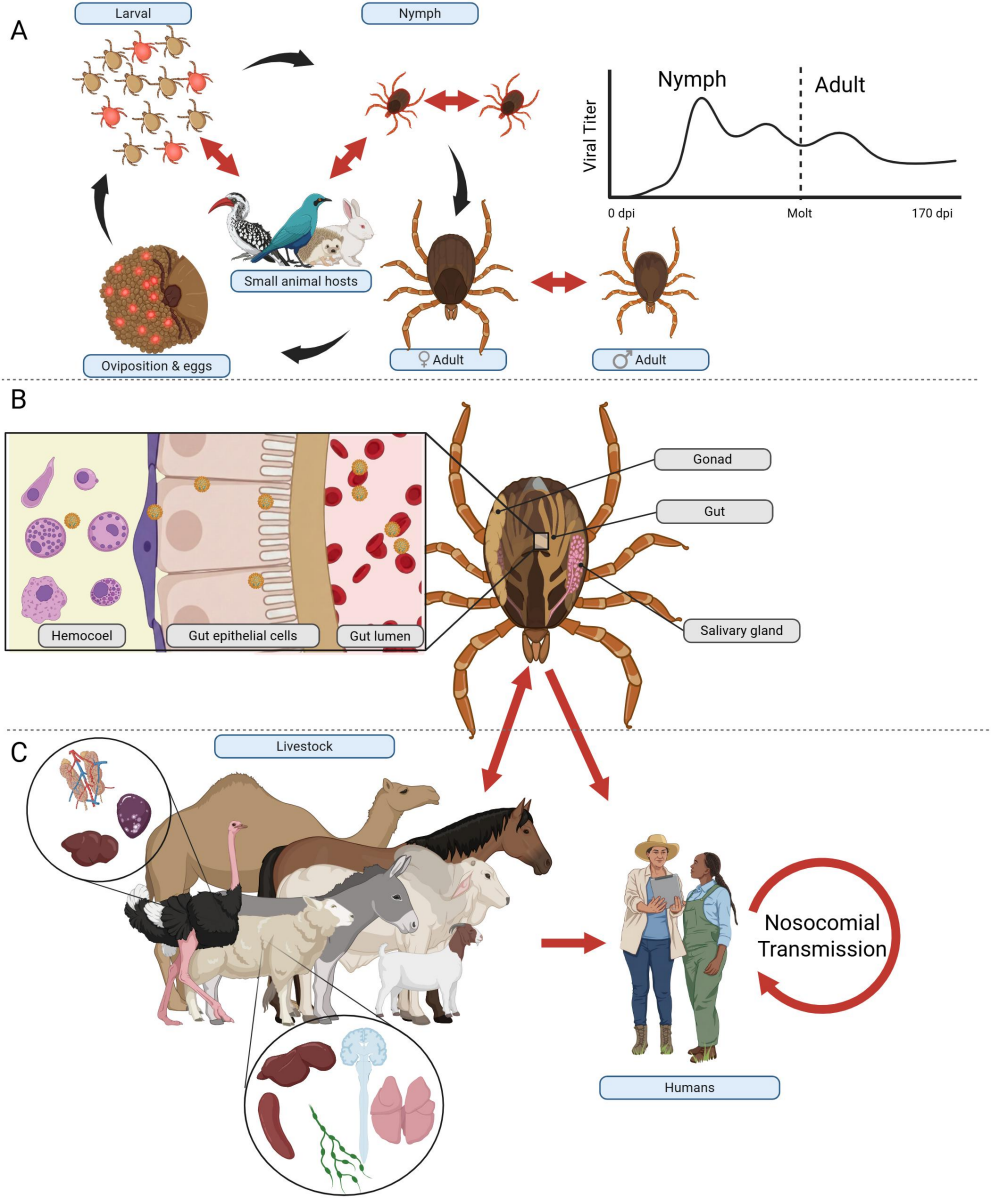
The zoonotic cycle of CCHFV within ticks (vector/reservoir), animals (host), and humans (host). (A) The transstadial and transovarial transmission of CCHFV in *Hyalomma* ticks, and transmission within and between adult ticks (venereal or co-feeding). The viral titer trends observed across tick life stages (in days) adapted from Shepherd et al., 1989 [27]. Small mammal and bird species which have been experimentally confirmed to be susceptible to and/or transmit CCHFV to larval or nymph tick stages. (B) Schematic illustration of CCHFV tissue tropism (gut, gonad, and salivary glands). (C) The vertebrate transmission chain between wild/domestic animal species and humans. Animals portrayed are examples of those identified to be susceptible to and/or transmit CCHFV. Call-outs: organs (Ostrich: kidney, liver, spleen; sheep: liver, spleen, lymph nodes, cerebrospinal fluid, lungs) in which CCHFV has been detected.

In the enzootic cycle of CCHFV, ticks acquire infection through horizontal transmission during a bloodmeal from a viremic vertebrate host or through co-feeding with infected ticks on a non-viremic host (Figure 1(A)) [30]. Based on observations from other tick-borne pathogens, CCHFV is thought to evade tick immune responses after acquisition during a bloodmeal, replicating in the midgut epithelium before disseminating through the hemolymph to additional tissues [31,32]. Although the kinetics of early infection events have not been directly demonstrated for CCHFV, viral antigen has been detected in hemocytes, salivary glands, and reproductive tissues of infected ticks supporting systemic dissemination (Figure 1(B)) [31,33–36].

Transmission can also occur vertically, when infection is passed from an adult female tick to her offspring (transovarial transmission [TOT]) [37]. In ixodid ticks, CCHFV can additionally be maintained across developmental stages (transstadial transmission) and transmitted between mating adults through venereal mechanisms (Figure 1(A)) [5,27,28,37].

Despite the capacity for transstadial and transovarial maintenance, infection prevalence and viral titers vary substantially across developmental stages and physiological states. In early vector competence studies, CCHFV was detected in only one of six larval pools, and virus was not subsequently recovered from nymphs that molted from those infected egg batches [37]. Similarly, larvae infected through blood feeding on viremic hosts exhibited low overall infection rates throughout the life cycle (4.4%), with stage-specific detection rates of 1.7% in larvae, 8.2% in unfed nymphs, and 3.1% in adults [38]. In contrast, *H. marginatum* nymphs fed on CCHFV-infected STAT-1^−/−^ mice, a model susceptible to CCHFV infection and disease, demonstrated 100% infection rates both after feeding and following molting [36]. This exceptionally high rate of transstadial transmission, compared with historical observations, may reflect the higher and more sustained viremia achieved in the immunodeficient STAT-1^−/−^ host [36].

Experimental intracoelomic infection studies in multiple species (*H. marginatum, H. rufipes, H. truncatum, Rhipicephalus evertsi mimeticus, and Amblyomma hebraeum*) further demonstrate stage-associated shifts in viral kinetics. Nymphs reach peak titers within the first week post-infection; however, titers decline prior to molting, and a modest rebound occurs upon adult emergence (Figure 1(A)) [27]. In infected adults, viral titers progressively decrease but remain detectable for several months, indicating the potential for long-term viral persistence [27,39]. Importantly, this persistent transstadial association is not a passive process for the virus. Passage through tick developmental stages imposes significant selective pressures that drive CCHFV genetic plasticity, resulting in consensus-level mutations not observed during mammalian infection [40]. Blood feeding may transiently increase viral loads, particularly in females, suggesting physiological modulation of replication during hematophagy [38,39]. In contrast to *Hyalomma* spp., *Amblyomma variegatum* exhibits more stable early-phase titers with less pronounced amplification, highlighting potential species-specific differences in replication dynamics [35].

Effects of pathogens on ticks have largely been inferred from changes in behavioral or physiological fitness. Some pathogens have a beneficial effect on their tick hosts’ fitness including increased phototaxis, upregulation of tick histamine release factor to improve blood flow at the bite site [41,42], increased activity and host-seeking behavior, greater tolerance to the insect repellent [43–45], improved resilience in cold environments [46], and desiccation tolerance [47,48]. In contrast, some pathogens have a negative effect on host fitness including malformations and impaired feeding behavior [49], reduced mobility and lower questing height [41], and reduced longevity or fecundity [50,51].

CCHFV infection in ticks appears to cause minimal physiological disruption. The virus can persist throughout all developmental stages and across generations and has not been shown to affect tick feeding success or alter egg production [24,52]. Experimental studies indicate that viral replication in ixodid ticks varies across generations, similar to the variation observed across developmental stages (Figure 1(A)). For example, in *H. truncatum*, TOT has been reported to range from 0–50% of egg batches tested [27,37,38,52]. This variability may reflect differences in inoculation route or viral strain. TOT rates of 50% were observed following intra-anal inoculation with strain HD49199 [37], whereas no transmission (0%) was detected after intracoelomic inoculation with strain SPU4/81 [27]. Intermediate transmission rates (17%) were reported when ticks acquired strain IbAr10200 through blood feeding on an experimentally infected host [52]. Notably, these values represent the proportion of positive egg batches rather than the percentage of infected eggs within each batch, which may obscure the true efficiency of transmission at the individual egg level.

Despite evidence of persistent infection, the gross and microscopic pathology associated with CCHFV infection in ticks remains poorly characterized [53,54]. Electron microscopy has been used to visualize pathogens within tick tissues, but these approaches have rarely been applied to assess tick pathology directly [55–58]. Only recently have standardized paraffin-embedded whole-tick fixation methods been developed to facilitate histopathological analysis [53]. Consequently, much of the current understanding of pathogen-induced cellular alterations in ticks derives from studies using tick-derived cell lines.

CCHFV can establish persistent infection in tick-derived cell lines, including HAE/CTVM9 and HAE/CTVM8 cells, heterologous embryonic cell lines derived from *H. anatolicum anatolicum* embryos [59]. Several other ixodid tick cell lines support CCHFV replication, although viral titers are generally lower than those observed in mammalian cell lines and infection does not produce a cytopathic effect [60]. Comparable observations have been reported for Hazara virus (HAZV), a closely related orthonairovirus, which induces apoptosis in mammalian cells but not in tick HAE/CTVM9 cells [61]. Recent studies have provided mechanistic insight into this persistence, demonstrating that both CCHFV and HAZV generate endogenous virus-derived DNA (vDNA) forms within tick cells. These findings suggest that vDNAs may play an important role in the establishment and maintenance of persistent, non-cytopathic infection in tick cells [62].

Recent studies of pathogen-tick interactions suggest that infection may nonetheless influence tick cellular physiology. Transcriptional profiling has demonstrated shifts in hemocyte gene expression during hematophagy, a physiologically stressful process, and in response to microbial challenge [63]. For example, infection with *Anaplasma phagocytophilum* or *Borrelia burgdorferi*, the bacterial agents of granulocytic anaplasmosis and Lyme disease, respectively, induces a metabolic shift toward glycolysis and broader remodeling of cellular metabolic pathways in ticks, similar to metabolic reprogramming described in other arthropods and vertebrates [64]. In contrast, *Rickettsia buchneri*, a tick endosymbiont, has minimal impact on host cellular bioenergetics. Whether CCHFV infection similarly alters tick metabolic pathways remains unclear. Together, these findings support the concept that CCHFV infection in ticks causes limited cellular pathology, although comprehensive *in vivo* characterization is still lacking.

### Intermediate vertebrate hosts

Numerous studies highlight the central epidemiological role of vertebrates in sustaining CCHFV transmission by serving as intermediary and amplifying hosts [27,52,65–68]. Immature *Hyalomma* ticks parasitize small mammals and birds, whereas adults feed primarily on ungulates (Figure 1(A–C)) [69]. A wide range of wild and domesticated vertebrates, including hares, ground squirrels, hedgehogs, tortoises, ungulates, and equids, have been implicated in maintaining the virus [13]. In these hosts, infection typically results in transient viral replication followed by short-lived viremia and seroconversion [66–68,70–77]. Due to this brief viremia period, evidence of active infection in vertebrate host species can be difficult to detect, even in areas with documented CCHFV circulation in ticks. One field study evaluated blood and milk samples from cattle, sheep, and goats during surveillance activities. Viral RNA (vRNA) was not detected in either blood or milk despite evidence of seroconversion in over 66% of animals tested and infection in ticks collected from the same region [78]. The absence of detectable vRNA may reflect the narrow temporal window of viremia and viral shedding, the possibility that viral loads were below the limit of detection, or intermittent viral presence. However, transmission to feeding ticks can also occur in the absence of detectable viremia, suggesting that low-level or localized infection may still contribute to viral maintenance in nature [79].

CCHFV infection in vertebrate hosts rarely produces overt clinical disease. Consistent with this observation, bovine cells are less permissive to infection, sustain less cellular damage, and produce lower viral yields than human cell lines [80]. Nevertheless, several studies suggest infection may not be entirely biologically silent. Ground squirrels developed self-limiting immunomorphological changes within the reticulohistiocytic system despite the absence of clinical signs [81]. Fever has been reported in horses, although attribution to CCHFV or components of the inoculum remains uncertain [75]. Young calves have been described as exhibiting lethargy and decreased appetite [68], while infected sheep developed mild fever, moderate increases in serum aspartate aminotransferase (AST), and marked neutrophilia [74].

Most experimental studies in vertebrate hosts have focused primarily on viremia and seroconversion, leaving tissue tropism and pathological sequelae relatively understudied. In sheep experimentally infected with the Kosovo Hoti strain, vRNA was detected in multiple tissues, including lymph nodes, liver, spleen, lung, and cerebrospinal fluid, up to 34 days post infection, indicating widespread viral dissemination despite failure to recover infectious virus (Figure 1(C) inset) [82]. Despite this dissemination, animals did not develop severe clinical disease but exhibited measurable physiological responses, including fever, a transient decline in neutrophils, and sustained lymphocytosis. Cytokine profiling further suggested suppression of pro-inflammatory signaling, with downregulation of interleukin-8 (IL-8), IL-4, and vascular endothelial growth factor (VEGF-A) with only limited increases in tumor necrosis factor alpha (TNF-α) and IL-1β. These responses may help limit vascular dysfunction and excessive neutrophil recruitment, processes associated with severe human disease (see System findings and anatomic pathology; See immune dysregulation and hyperinflammation, cytokine dysregulation, and endothelial dysfunction and hemostatic failure within Cell entry and molecular determinants of disease). Observations in other vertebrate hosts are consistent with this pattern of viral dissemination with limited pathology; infectious virus has been isolated from the liver and spleen of experimentally infected scrub hares [71], and viral RNA detected in the spleen, liver, and kidney of infected ostriches without associated histopathological lesions (Figure 1(C) inset) [76].

In contrast to the largely subclinical phenotype of CCHFV infection in ruminants, other orthonairoviruses produce markedly different disease outcomes in the same host background. Nairobi sheep disease virus (NSDV; also known as Ganjam virus in India), a closely related ixodid-transmitted virus, causes severe and frequently fatal hemorrhagic gastroenteritis in sheep with widespread viral dissemination [83–85]. Mechanistic studies suggest that differences in host immune modulation may underlie this divergence. NSDV infection induces rapid transcriptional upregulation of inflammatory cytokines in sheep [86], and its ovarian tumor (OTU) protease delays interferon-β (IFN-β) activation and suppresses type I and II IFN signaling [87]. More recent studies indicate that NSDV also induces autophagy, whereas inhibition of this pathway enhances IFN responses and restricts viral replication [88].

Together, these observations suggest that virulence in ruminants is not determined solely by systemic viral dissemination but rather by differences in host-virus immune interactions. Species-specific interactions between viral OTU domains and host IFN-stimulated gene-15 (ISG15) may be particularly relevant, as *Orthonairovirus* OTU domains exhibit differential specificity for ISG15 from different mammalian species [89]. Even single amino acid (AA) substitutions within the OTU substrate-binding interface can markedly alter cleavage efficiency toward human or sheep ISG15, suggesting that subtle structural differences may influence IFN antagonism and, consequently, pathogenic potential [89].

## Pathogenicity and virulence in humans

### Clinical symptoms

In humans, CCHF has a variable incubation period that depends on the route of exposure [90]. Infection via tick bite is typically associated with a shorter incubation period of one to seven days, which, like other tick-borne pathogens, is thought to be due to saliva-assisted transmission [91–93]. Exposure to blood or tissue from infected animals is associated with a longer incubation period of approximately five to fourteen days [10,90,94–97]. While incubation periods typically do not exceed two weeks, longer incubation periods have also been reported [98,99].

**Figure 2.**
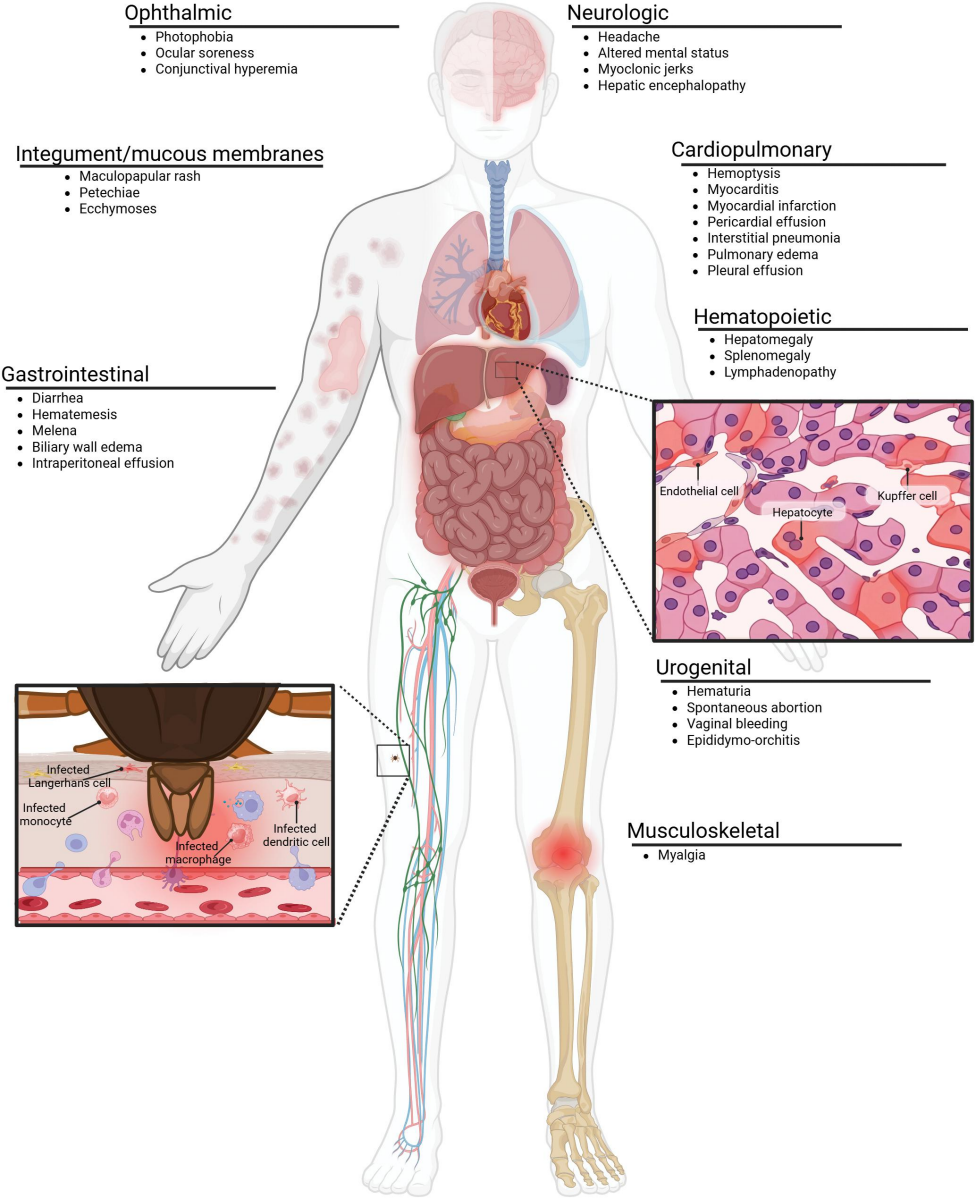
Clinical manifestations of CCHF in humans by organ system following tick-borne exposure. Summary of reported clinical signs and symptoms associated with CCHF infection, organized by organ system. Left inset: cell types known to be susceptible to CCHFV following natural exposure (tick bite). Right inset: representative susceptible hepatic cell populations within the liver, a well-established target organ of CCHFV infection.

The clinical spectrum of CCHF ranges from mild illness to fatal disease. Common symptoms are illustrated in Figure 2. Following the incubation period, illness typically begins with a pre-hemorrhagic phase lasting one to seven days and characterized by nonspecific symptoms including fever, headache, nausea, vomiting, abdominal pain, photophobia, eye soreness, myalgia, and/or generalized body aches [90,100]. In some patients, the disease progresses three to five days after symptom onset to a hemorrhagic phase marked by a maculopapular rash and petechiae involving mucous membranes, which may advance to more severe manifestations such as ecchymoses, epistaxis, hemoptysis, hematemesis, diarrhea, melena, and hematuria [5]. Severe cases develop a systemic illness affecting multiple organs. Renal insufficiency, DIC, and shock are common complications associated with severe disease [5,90,100,101].

Well-characterized clinical laboratory abnormalities have been incorporated into disease severity scoring systems, particularly markers reflecting hepatic injury and coagulopathy (Supplemental Table 1). Indicators associated with terminal disease include thrombocytopenia (<20,000/mm^3^), markedly elevated liver enzymes (AST >700 U/L and/or alanine transaminase [ALT] >900 U/L), and evidence of severe coagulopathies, such as activated partial thromboplastin time (aPTT) >60 seconds or fibrinogen <110 mg/dL) [90,95,102,103]. In addition to these system-specific abnormalities, elevated markers of generalized tissue injury and inflammation, including lactate dehydrogenase and creatine phosphokinase, are frequently observed [104,105]. Organ-specific laboratory abnormalities are discussed in subsequent sections. Most deaths occur during the second week of illness and are associated with refractory shock, multiorgan failure, severe coagulopathy, and acute hepatopathy [5,106].

### System findings and anatomic pathology

#### Hematologic and reticuloendothelial system

**Figure 3.**
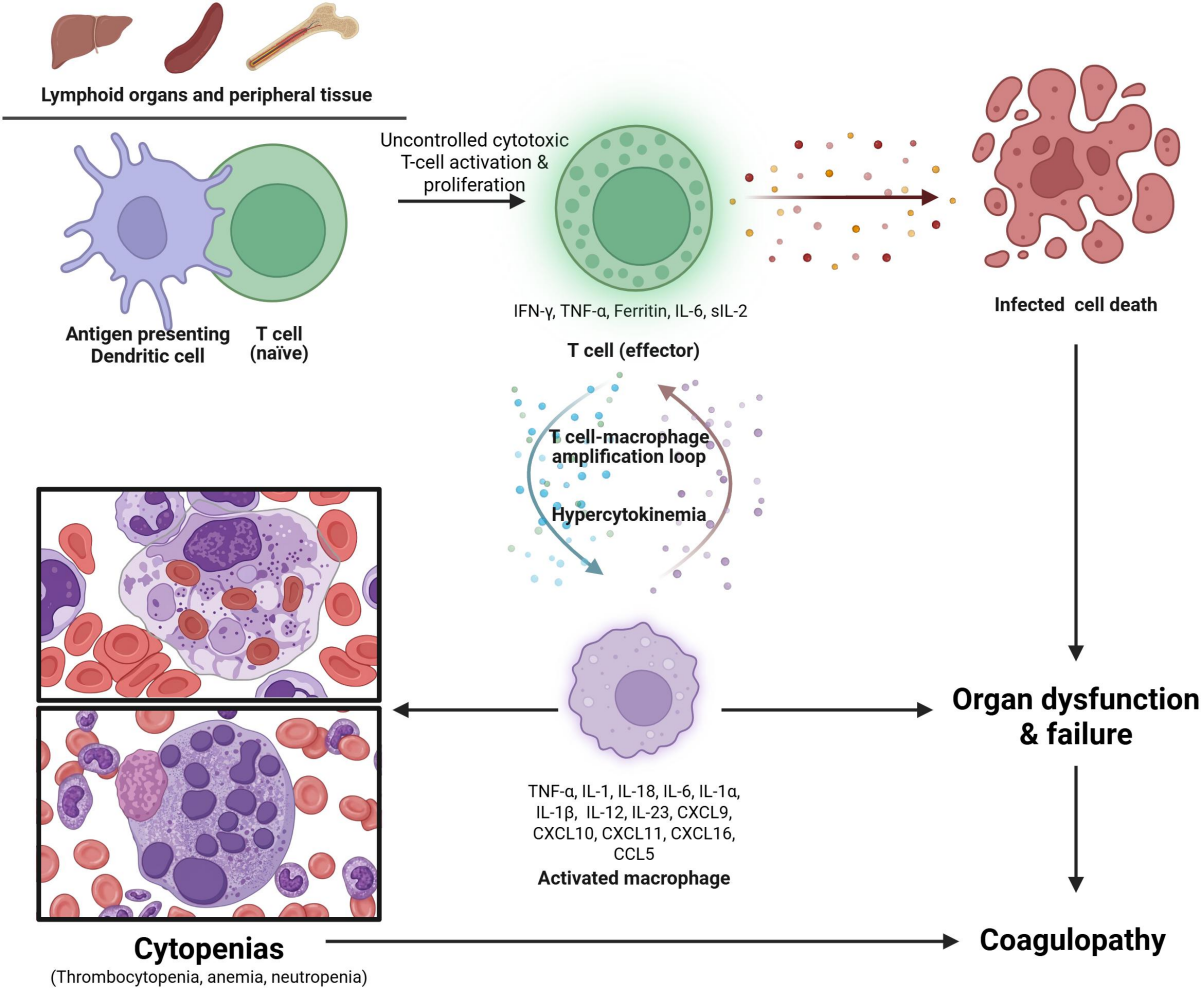
CCHFV-induced macrophage activation syndrome (MAS) or hemophagocytic lymphohistiocytosis (HLH)-like syndrome. Lymphoid and peripheral tissues commonly affected by MAS include the liver, spleen, and bone marrow. Antigen-presenting cells (dendritic cells) activate T-cells, which facilitate apoptosis and activate macrophages. Inflammatory dysregulation associated with CCHFV results in a hyperinflammatory syndrome characterized by excessive innate immune activation and hypercytokinemia. Apoptosis results in organ dysfunction and failure. The heightened immune response can also result in hemophagocytosis within the spleen and bone marrow resulting in cytopenias. Lower left top panel: an activated macrophage that has phagocytosed platelets and red blood cells within the spleen. Lower left bottom panel: an activated macrophage that has engulfed hematopoietic precursor cells within the bone marrow. The combination of cytopenias, specifically thrombocytopenia, and organ dysfunction and/or failure can result in coagulopathies due to destruction of platelets peripherally or low production of platelets in the bone marrow and altered production of coagulation factors by the liver.

CCHFV prominently affects the hematopoietic and reticuloendothelial systems, including the bone marrow, spleen, lymph nodes, and liver. Hematologic abnormalities are a hallmark of disease and include thrombocytopenia and leukopenia. Bone marrow findings reveal microthrombi, hemorrhages, and hemophagocytosis [107,108]. Splenic pathology includes prominent lymphoid apoptosis and marked lymphocyte depletion, as well as splenomegaly and lymphadenopathy documented sonographically and by computed tomography (CT) [109–111]. Changes in circulating leukocyte populations also correlate with disease severity. Neutrophilia accompanied by lymphopenia and monocytopenia at admission has been associated with mortality, although leukocytosis has also been observed in fatal cases [112]. More recently, cellular profiling of severely ill patients demonstrated increased immature granulocyte populations, hyperactivated neutrophils, bone marrow stress, immune exhaustion, and dysregulation of lymphocyte populations, whereas mild disease was associated with more controlled immune responses and greater lymphocyte activity [113]. Macrophage activation syndrome (MAS) and reactive hemophagocytic lymphohistiocytosis (HLH) have increasingly been recognized in CCHF patients and contribute to pancytopenia (Figure 3) [114]. HLH has been associated with more severe disease in children [115–117] and has also been reported in adults [118] (see Monocyte/Macrophage activation, hemophagocytosis, and T-cell dysfunction). Similar trends have been observed in the transiently immunosuppressed murine model of CCHF, in which animals infected with strain Turkey-2004 developed mild disease characterized by early neutrophil and macrophage recruitment followed by lymphocytic infiltration, whereas infection with IbAr10200 resulted in sustained inflammatory responses, lymphocyte necrosis or apoptosis, and fatal disease [119].

#### Hepatic system

Hepatic involvement is another defining feature of CCHF pathogenesis. Radiologic studies frequently demonstrate hepatomegaly regardless of disease severity (Supplemental Tables 2 and 3) [110,120,121]. Elevated AST and ALT, consistent with a hepatocellular injury pattern, are also common findings (Supplemental Table 1). Severe cases may progress to hepatic encephalopathy or acute hepatic failure [122]. Vascular complications, including thrombus formation in the central and portal veins and portal vein thrombosis, have been associated with severe hepatic involvement [90,118]. Histopathologic lesions include eosinophilic necrosis, Kupffer cell hyperplasia, and the presence of “Councilman bodies,” reflecting infection of mononuclear phagocytes, endothelial cells, and hepatocytes that play central roles in disease pathogenesis (Figure 2, middle right inset) [109]. Additional hepatic findings include cytoplasmic macrovesiculation and microvesiculation, erythrocyte infiltration with diffuse extravasation, pericapillary edema, hepatocyte autolysis, and intracytoplasmic virions within hepatic sinusoidal and portal epithelial cells [107,123].

#### Cardiopulmonary system

Cardiopulmonary involvement in CCHF reflects both systemic inflammatory responses and vascular injury. Cardiac manifestations include myocarditis, myocardial infarction, and pericardial effusion (Figure 2). Echocardiographic studies demonstrate that patients with severe disease often exhibit mildly reduced left ventricular ejection fraction, elevated systolic pulmonary pressures, and pericardial effusions [124]. Pediatric cases can present with transient cardiac dysfunction, including mild pericardial effusion and reversible wall motion abnormalities [125,126]. Electrocardiographic abnormalities such as T-wave inversion and bundle branch block occur more frequently in fatal cases and may serve as prognostic indicators [127].

Pulmonary manifestations include interstitial pneumonia, pulmonary edema, pleural effusion, and ground-glass opacities (Figure 2) [109,110,120]. In a CT-based imaging study of 112 CCHF cases, non-survivors exhibited higher frequencies of pleural effusion, atelectasis, lung consolidation, and pericardial effusion [128]. Supporting these findings, elevated cardiac troponin T, a biomarker of myocardial injury, has been detected in severely ill CCHF patients presenting with hemorrhage [129].

#### Gastrointestinal system

Gastrointestinal involvement reflects both mucosal injury and systemic vascular pathology. Histopathologic examination has demonstrated complete epithelial denudation of the colon [107]. Imaging studies frequently report gallbladder wall thickening and intraperitoneal effusion (Supplemental Tables 2 and 3, Figure 2) [110,120]. Free intraperitoneal fluid has been significantly associated with coagulopathy and, together with gallbladder wall abnormalities, correlates with increased mortality [120].

#### Reproductive system

Reproductive tract manifestations of CCHFV infection are infrequently described and remain poorly characterized. Most available clinical observations associate infection during pregnancy with adverse fetal outcomes, including spontaneous abortion, premature birth, and fetal mortality [130,131]. In one case, vRNA was detected in uterine tissue obtained after a spontaneous abortion, suggesting potential viral involvement in adverse pregnancy outcomes. Notably, the tissue sample was collected nine weeks after resolution of acute disease, raising the possibility of viral persistence in reproductive tissue [132]. Although most reports describe poor fetal outcomes, a small number of cases have documented uncomplicated live births following maternal CCHFV infection [130,131].

Non-gynecologic vaginal bleeding (Supplemental Table S3, Figure 2) has been reported as a presenting manifestation of CCHF in both retrospective cohort studies and case reports [133–135]. In males, two cases of epididymo-orchitis temporally associated with acute CCHF have been described, suggesting that inflammatory involvement of the testis or epididymis may occur during infection [136,137].

#### Neurologic system

Neurologic and neuropsychiatric manifestations in CCHF are reported but appear relatively uncommon and are most frequently observed during the hemorrhagic phase of disease. Reported symptoms include agitation, confusion, delusions, neck stiffness, headache, photophobia, and, more rarely, myoclonic jerks (Figure 2) [138]. Severe intracranial complications are rare and have been described primarily in isolated cases, including one case of acute subdural hematoma and three cases of cerebral hemorrhage. These events are generally attributed to severe thrombocytopenia rather than direct viral invasion of the central nervous system [90,139].

Consistent with this interpretation, a prospective study of CCHF patients found no magnetic resonance imaging (MRI) abnormalities or evidence of encephalitis despite the presence of neurologic symptoms, suggesting that many manifestations reflect systemic complications such as hepatic encephalopathy rather than primary CNS infection [138]. Confirmed viral encephalitis appears exceedingly rare, with only one documented case to date [140] and a single report of virus isolation from the hypothalamus of a deceased patient [141]. Overall, available evidence indicates that neurologic findings in CCHF are more likely secondary to systemic pathophysiology than indicative of primary viral neurotropism.

#### Ophthalmic system

Ophthalmic manifestations of CCHF are generally mild and transient but reflect systemic vascular involvement. Conjunctival hyperemia is commonly observed and has been reported in approximately half of patients (Figure 2) [142,143]. Fundoscopic abnormalities, including dilated retinal veins and increased retinal vessel tortuosity, have been described in pediatric cases, although these findings did not differ significantly across disease severity groups [142]. Spectral-domain optical coherence tomography studies have demonstrated increased choroidal thickness in CCHF patients compared with healthy controls, although the differences were not statistically significant [144]. These findings nevertheless suggest structural and vascular alterations potentially associated with endothelial injury, vascular leakage, and inflammation [145]. One case of self-limiting post-infectious retinal involvement consistent with Purtscher-like retinopathy has also been reported [146].

## Cell entry and molecular determinants of disease

The CCHFV genome consists of three single-stranded RNA segments, termed small (S), medium (M), and large (L) based on their relative sizes. The bipolar S segment encodes the viral nucleoprotein (NP) and a non-structural protein (NSs) in opposite reading frames; however, unlike canonical ambisense segments, the NP and NSs open reading frames overlap and are not separated by a defined intergenic region [147–149]. The M segment encodes the glycoprotein precursor (GPC), which undergoes post-translational cleavage to generate the mature envelope glycoproteins Gn and Gc, as well as the non-structural protein NSm. The L segment encodes the multifunctional L protein, which contains several functional domains, including the viral RNA-dependent RNA polymerase (RdRP), an N-terminal OTU protease, and an endonuclease involved in cap-snatching and RNA processing [147,150–154].

### Cell entry and replication

Viral entry into host cells is mediated by the Gc glycoprotein, which binds to host cell factors and triggers cholesterol- and pH-dependent clathrin-mediated endocytosis [155–157]. Earlier studies identified several putative attachment or accessory receptors, including nucleolin [158], DC-SIGN [159], and MFGE8 [160]. Heparan sulfate has also been implicated as an attachment factor that may facilitate viral binding to the cell surface [161]. More recently, the low-density lipoprotein receptor (LDLR) has been identified as a functional entry receptor for CCHFV [157,162,163].

LDLR-dependent entry appears to occur through complementary mechanisms involving direct virus-receptor engagement and host protein-mediated attachment. LDLR has been identified as an important CCHFV entry factor in multiple susceptible cell types and mouse models, with soluble LDLR, LDLR-targeting antibodies, and genetic disruption of LDLR reducing infection [162,163]. Mechanistically, LDLR can directly engage CCHFV glycoproteins, including high-affinity binding to Gc, thereby supporting viral attachment and internalization [162]. Apolipoprotein E (ApoE) provides an additional LDLR-dependent route of attachment. CCHFV particles can incorporate host-derived ApoE, which acts as a bridging ligand for LDLR and enhances particle binding and specific infectivity [157,163]. This ApoE-associated mechanism appears to distinguish CCHFV from the related Hazara virus, which does not incorporate ApoE into virions and does not utilize LDLR for productive entry [157,163].

The cellular distribution and regulation of LDLR may also influence tissue tropism and infection amplification. In polarized epithelial cells, CCHFV preferentially enters and exits from the basolateral surface, consistent with the basolateral localization of LDLR and other host entry factors [164–167]. Factors released from CCHFV-infected monocyte-derived dendritic cells have also been shown to enhance CCHFV infection of polarized cells, suggesting that inflammatory signaling may modulate viral entry or cellular permissiveness, although the specific mediators and their relationship to LDLR expression remain incompletely defined [166,168,169]. *In vivo*, the importance of LDLR is supported by studies demonstrating that LDLR deficiency or LDLR blockade reduces viral burden, pathology, and disease severity and delays disease progression in mouse models of CCHFV infection [162,163]. Notably, whereas transiently immunosuppressed wild-type mice uniformly succumb to infection, similarly treated *Ldlr*^−/−^ mice exhibit delayed onset of clinical disease, reduced viral burden, and substantially improved survival, with mortality reduced to 50% in one study [162,163].

The relative contribution of direct glycoprotein-LDLR binding compared to ApoE-mediated attachment likely depends on both cell type and the source of the virion. Virus produced in tick cells, where ApoE incorporation is not expected, can still utilize LDLR-dependent entry through direct interactions between viral glycoproteins and LDLR [163]. By contrast, virus produced in mammalian cells may exploit both direct LDLR engagement and ApoE-enhanced LDLR-dependent attachment [157,163]. Whether LDLR plays an equivalent role in natural ungulate hosts remains unresolved, and available data suggest that alternative or redundant entry pathways may contribute to CCHFV infection in these reservoir species [157].

Following receptor-mediated uptake, virions traffic through early endosomes to multivesicular bodies, where low pH triggers membrane fusion and release of ribonucleoprotein complexes into the cytoplasm. The viral L protein then transcribes capped viral messenger RNAs (mRNAs) from the negative-sense genomic segments for translation. In parallel, genome replication proceeds through synthesis of a full-length complementary positive-sense antigenome, which serves as the template for generation of new negative-sense genomic RNA segments [147].

The GPC is translated in the endoplasmic reticulum (ER) and undergoes extensive proteolytic processing during trafficking through the ER, Golgi apparatus, and trans-Golgi network. This processing ultimately produces the mature structural glycoproteins Gn and Gc along with several non-structural proteins, including NSm, GP160, GP80, and GP38 [147]. Newly synthesized genomes are packaged into enveloped particles that bud into the Golgi apparatus and are subsequently released from the cell via the secretory pathway [10,147,170].

### Cellular tropism and early dissemination

Natural CCHFV infection most commonly occurs through tick bite, resulting in dermal inoculation of virus into the skin. At the site of entry, CCHFV encounters multiple susceptible cell types within the dermis (Figure 2, lower left inset) [147,171–173]. *In vitro* and *in vivo* studies demonstrate that several subsets of peripheral blood mononuclear cells (PBMCs), including monocytes, monocyte-derived macrophages, and monocyte-derived dendritic cells (DCs), are permissive to infection, although productive replication varies substantially among cell types [172,174,175]. Dermal antigen-presenting cells, including dermal DCs and Langerhans cells, are also susceptible to infection, though replication efficiency varies depending on both cell subtype and viral strain [171,172,174–177]. This heterogeneity in permissiveness likely reflects a combination of strain-specific differences in viral entry or replication and host cell-intrinsic factors (see Viral strain variability and differences in virulence) [119,147,175]. In contrast, lymphocytes appear largely refractory to infection, suggesting that CCHFV preferentially targets cells of the monocyte-macrophage and dendritic cell lineages rather than T, B, or NK cells [172,174,175]. Productive infection of these cells likely facilitates early local amplification and dissemination. Infected antigen-presenting cells can traffic to regional lymph nodes, while infection of circulating monocytes may support hematogenous spread and systemic dissemination [109,172,174,178,179].

Although tick bite represents the primary route of natural infection, additional transmission routes have been documented. Percutaneous exposure, such as needlestick injuries sustained while handling infected livestock or caring for CCHF patients, as well as mucosal exposure to infectious blood or bodily fluids during animal slaughter or clinical management of infected individuals, can also result in infection [16,180,181]. Experimental animal models have therefore employed intradermal or subcutaneous, intramuscular, intranasal, and intraperitoneal inoculation routes to model these exposures [119,177]. These routes generally produce comparable systemic disease phenotypes, although differences in disease onset suggest that early infection dynamics may vary depending on the route of exposure [177]. To date, however, no controlled head-to-head studies have directly compared tick-bite infection with experimental inoculation routes to determine how these exposures influence early tissue tropism, immune priming, or subsequent pathogenesis.

Following local replication, CCHFV is thought to disseminate hematogenously and establish infection in multiple organ systems, most prominently the liver, spleen, and vascular endothelium [176,178,182]. In both humans and animal models, infection demonstrates pronounced hepatotropism, with viral antigen detected in Kupffer cells, hepatic endothelial cells, and hepatocytes (Figure 2, middle right inset). These findings are consistent with the central role of the liver in viral replication, immune dysregulation, and coagulopathy during severe disease [109,177,183]. Endothelial infection has also been documented in multiple organs, including the adrenal glands, using immunohistochemistry and in situ hybridization [109,174]. Histopathologic analyses of fatal human cases reveal marked splenic lymphoid depletion, dilated sinusoids, and lymphoid apoptosis, reflecting extensive involvement of the reticuloendothelial system [23,109,174,178,179,183]. While additional organ involvement, including heart and lung infection, has been described in animal models with compromised IFN signaling [177], direct evidence of productive infection in these tissues in humans remains limited [109].

Neuroinvasion has been observed in some experimental systems but appears uncommon. In one atypical humanized mouse model that did not reproduce the clinical course observed in standard acute lethal models, glial cells within the central nervous system were identified as principal targets of infection in terminal animals [184]. Detection of viral antigen in glial cells represented the only distinguishing pathological feature between lethal infection with strain Turkey-2004 and nonlethal infection with strain Oman-1998 and was accompanied by central nervous system manifestations including gliosis, meningitis, and meningoencephalitis [184]. These findings are consistent with rare reports of neurologic manifestations in human cases [95,138,140], although CNS infection is not consistently observed in conventional lethal models [185].

The extent and clinical significance of reproductive tract infection remain poorly characterized. Current evidence is largely derived from isolated case reports describing reproductive organ involvement and studies detecting vRNA in vaginal or urethral swabs [21,107,132,186]. Experimental mouse models have demonstrated CCHFV tropism for reproductive tissues, with viral antigen detected in non-parenchymal tissues including the tunica vaginalis and testicular or epididymal interstitium in males and the ovarian, uterine, and oviductal stroma in females [187]. Infectious virus has also been recovered from urogenital swabs during acute infection, suggesting reproductive tract involvement and raising the possibility of transmission or persistence within these compartments. Persistent virus has additionally been detected in the testes of nonhuman primates, although interpretation of these findings may be complicated by concurrent latent tuberculosis infection in the study animals [188]. Collectively, these observations suggest that reproductive tissues may represent sites of viral replication during both acute and potentially convalescent phases of infection.

### Systems-level insights from transcriptomic and proteomic studies

While identifying cellular targets and patterns of viral dissemination is critical for understanding CCHFV pathogenesis, defining how infection of these cells drives the complex immunopathology observed in severe disease requires broader systems-level approaches. Transcriptomic and proteomic analyses have begun to provide a framework for understanding these host responses. Studies of whole blood [189–192] and PBMCs [193] from CCHF patients reveal widespread dysregulation of host pathways implicated in immune activation, inflammation, and vascular dysfunction.

Analyses of long non-coding RNA (lncRNA) expression profiles demonstrate extensive modulation of host immune regulatory networks, including pathways involved in IFN, NF-κB activity, and antiviral responses [189,192]. Similarly, microRNA profiling across multiple datasets identifies convergent alterations in pathways related to Toll-like receptor (TLR) signaling, inflammatory signaling cascades, innate immune responses, platelet activation and degranulation, vascular integrity, and cellular metabolic processes [190,191,193]. Collectively, these findings highlight coordinated perturbations across immune, endothelial, and metabolic networks during infection.

Despite these insights, current transcriptomic datasets provide limited mechanistic resolution. Changes in gene expression do not necessarily reflect protein abundance or activity and may not capture post-transcriptional regulation, cell type-specific effects, tissue-specific effects, or functional pathway activation. In addition, many observations remain to be experimentally validated. Consequently, these datasets should be viewed primarily as hypothesis-generating frameworks. Throughout the following sections, systems-level findings are revisited alongside targeted experimental or clinical studies to highlight convergent evidence and to place these broader host-response signatures within the context of defined mechanisms of CCHFV pathogenesis and virulence.

### Innate immune sensing and viral-mediated immune evasion

**Figure 4.**
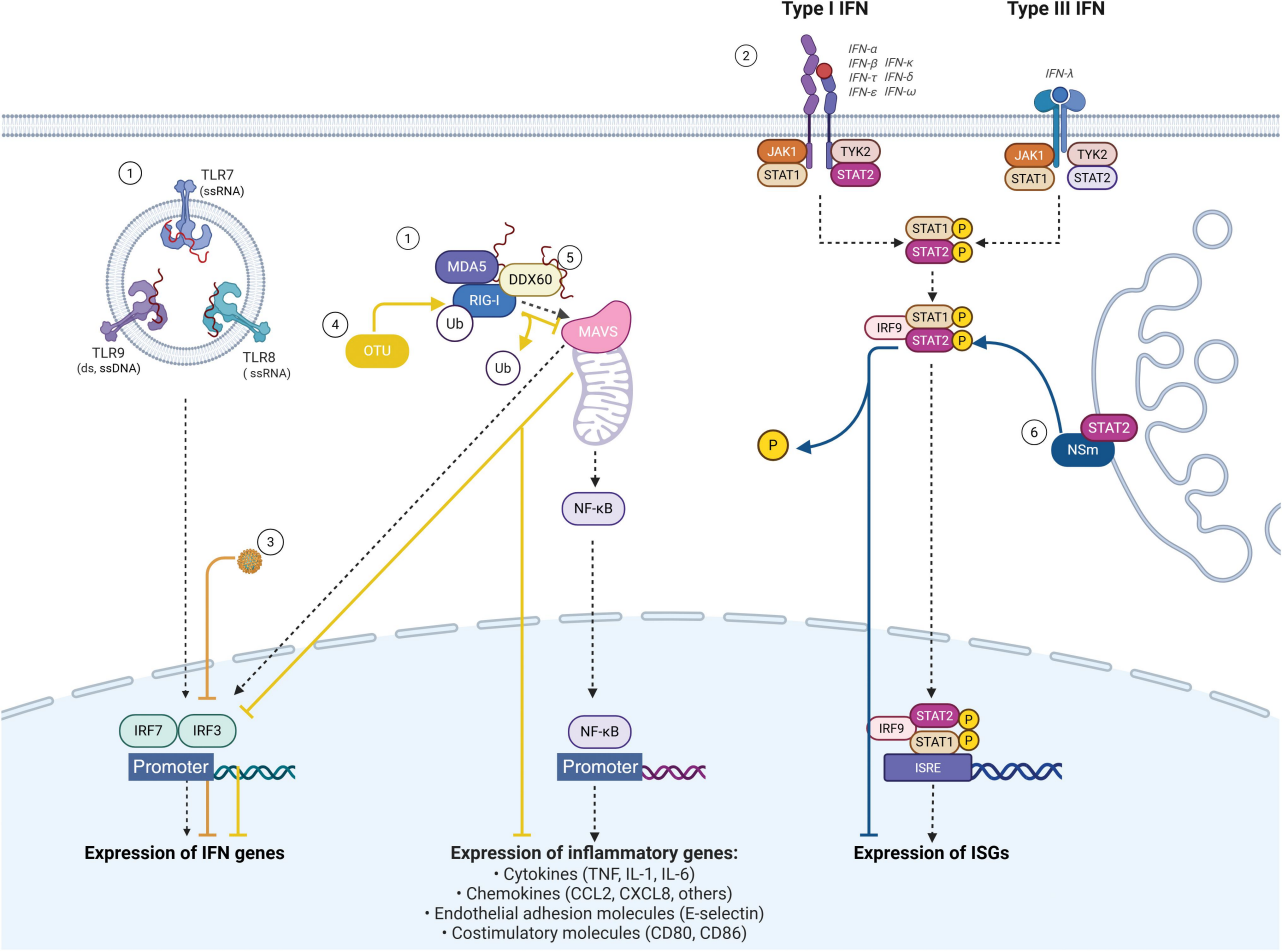
Innate immune sensing and identified viral-host interactions. Steps 1 and 2 depict key components of the host antiviral response. (1) CCHFV is recognized by Toll-like receptors (TLRs) and the cytosolic RNA sensor, retinoic acid-inducible gene I (RIG-I), triggering signaling cascades that induce the transcription of proinflammatory cytokines and antiviral genes. (2) Secreted type I interferons (IFNs) act on neighboring cells to stimulate the expression of interferon-stimulated genes (ISGs), establishing an antiviral state. Steps 3–6 illustrate distinct mechanisms by which CCHFV antagonizes host innate immune responses. These include: (3) delayed nuclear translocation of interferon regulatory factor 3 (IRF3), postponing IFN-α/β induction [195]; (4) antagonism of innate immune signaling by the viral ovarian tumor (OTU) protease domain through deubiquitination of host proteins, including RIG-I, and removal of ISG15 modifications from cellular substrates [198,199]; (5) interaction of the M segment-encoded glycoprotein precursor (GPC) mRNA with DDX60 to facilitate viral replication through unfolding of its G-quadruplex structure [201]; and (6) suppression of STAT-2 expression and phosphorylation by the nonstructural protein NSm, including relocalization of STAT-2 from the cytoplasm to the trans-Golgi network, thereby impairing IFN signaling [202].

Upon infection, CCHFV is recognized by host pattern-recognition receptors, such as TLRs, that initiate IFN signaling cascades. In addition, and despite the CCHFV genome being monophosphorylated, retinoic acid-inducible gene I (RIG-I) has been identified as an important sensor of vRNA and a key mediator of IFN induction during infection [194]. Activation of RIG-I promotes downstream signaling through mitochondrial antiviral signaling protein (MAVS), leading to transcription of IFNs and IFN-stimulated genes (ISGs), and inflammatory genes (Figure 4). Despite activation of these antiviral pathways, multiple studies indicate that CCHFV actively suppresses IFN signaling. *In vitro* experiments demonstrate that viral replication delays IFN production and remains largely resistant to the antiviral effects once IFN responses are established [195]. Consistent with this observation, exogenous IFN treatment inhibits viral replication only when administered prior to infection [195–197].

Several viral factors have been proposed to contribute to this IFN antagonism. The OTU-like cysteine protease domain encoded within the L protein contributes to antagonism through deubiquitinase and deISGylase activities that impair upstream antiviral signaling *in vitro* [198,199]. More recent studies have reported that CCHFV infection induces increased expression of DDX60, a helicase involved in the RIG-I signaling pathway, which the virus may exploit to facilitate vRNA unwinding and replication [200,201]. The nonstructural protein NSm has also been suggested to play a role in IFN antagonism through interactions with signal transducer and activator of transcription 2 (STAT-2) [202].

Antagonism of IFN signaling is widely considered a major determinant of severe CCHF in humans [175,203–206]. However, the precise role of IFN responses in disease progression remains complex and incompletely resolved. In clinical studies, elevated IFN-γ levels have been associated with poor outcomes [207]. In contrast, experimental studies in nonhuman primates and mouse models suggest that IFN-γ contributes to viral control, as blockade of IFN-γ signaling increases viral loads and mortality [182,208,209]. Similarly, *in vitro* studies indicate that type I and type III IFNs (IFN-α and IFN-λ1) each exert antiviral effects but may display antagonistic interactions when combined [210].

Systems-level transcriptomic analyses further highlight the complexity of IFN regulation during CCHFV infection. Altered microRNA signatures targeting IFN induction and IFN regulatory factor (IRF) signaling pathways have been reported in patient samples [190]. In addition, the long non-coding RNAs negative regulator of IFN response (NRIR) and negative regulator of antiviral response (NRAV) have been found to be significantly elevated in fatal cases [189,192,211]. Paradoxically, IFN-stimulated genes such as interferon-induced transmembrane proteins (IFITM1 and IFITM3), which are downstream targets of these regulatory networks, are upregulated in both human patients and nonhuman primates experiencing mild or nonfatal disease [189,192,211]. These cases also exhibit increased expression of ISGs including Mx1, Mx2, and OAS1–3, suggesting the presence of a more intact antiviral response [200,212–214].

Taken together, these observations indicate that IFN responses during CCHFV infection are highly dynamic and context dependent. Apparent discrepancies across studies likely reflect differences in disease stage, sampling time points, organ- and system-specific responses, and cell type-specific effects. Consequently, the temporal dynamics of IFN signaling and its relationship to disease severity remain areas of active investigation.

### Viral strain variability and differences in virulence

Corresponding with its wide geographic distribution, CCHFV exhibits substantial genetic diversity, likely reflecting selective pressures within tick reservoirs and mammalian amplifying hosts rather than humans [40,215,216]. Multiple genotypes have been described based on geographic distribution. Genotypes I – III circulate predominantly in Africa, genotype IV in Asia, and genotype V in eastern Europe, Kosovo, and Türkiye [148,216,217]. A sixth lineage previously classified as Europe II (AP-92) has recently been reclassified as a distinct virus, Aigai virus (*Orthonairovirus parahaemorrhagiae*) [218–220], and an additional genotype (VII, Africa) has been proposed [216,221]. Although strains within a given region tend to remain relatively stable over time, long-distance dispersal through migratory birds carrying infected ticks and genome segment reassortment contribute to global lineage mixing and genetic diversification [148,222–226].

Whether this genetic diversity directly contributes to the variable clinical course of human CCHF remains uncertain. The most compelling evidence for strain-associated differences in pathogenicity derives from Aigai virus, a virus once considered CCHFV but now technically a related virus [220]. In Greece, this lineage is associated with appreciable human seroprevalence despite an absence of recognized clinical disease, suggesting substantially reduced pathogenicity compared with other CCHFV lineages [227,228]. To date, only a single fatal human infection has been reported in Iran [229].

Molecular evidence supporting strain-specific differences in infectivity has also been reported. A Europe II tick isolate (MT-1303) demonstrated reduced capacity to infect human cells, associated with a single AA substitution within the PreGc/Gc region of the viral glycoprotein [230]. This strain uniquely encodes a glycine at position 1116 (G1116), whereas other strains contain a conserved arginine at this position (R1116). Functional studies using viral-like particle reporter systems showed that restoring the conserved residue (G1116R) rescued infectivity to levels comparable with the Africa 3 strain IbAr10200, while introducing the reciprocal (R1116G) mutation into IbAr10200 reduced infectivity to that of MT-1303 [230]. These findings indicate that subtle glycoprotein differences may influence viral entry efficiency and potentially virulence.

Experimental animal models demonstrate strain-dependent variation in disease outcome, although interpretation is complicated by differences in viral stocks, passaging history, and experimental design [230]. In IFNAR^−/−^ mice, subcutaneous infection with IbAr10200 (Africa III) or Turkey-2004 (Europe I) produces uniformly lethal disease, whereas infection with Asia I isolates (UAE, Oman-1997, or Oman-1998) results in transient, self-limiting illness [231].

Comparative studies suggest that divergent clinical phenotypes among strains may be driven less by differences in viral replication or dissemination and more by variation in host inflammatory and hematopoietic responses. In the transiently immunosuppressed mouse model, infection with IbAr10200 results in uniformly lethal disease, whereas the Turkey-2004 strain causes a transient, self-limiting illness despite comparable viral loads and tissue distribution [119]. The lethal phenotype was associated with more robust systemic inflammatory responses and more severe hematopoietic pathology. Similarly, comparison of the lethal Asia I strain Afg09-2990 with the attenuated Kosovo Hoti strain demonstrated higher viremia following Afg09-2990 infection, comparable hepatic viral burden and liver pathology between strains, and markedly attenuated inflammatory cytokine responses in Hoti-infected animals [232]. Consistent with these findings, nonhuman primate studies comparing Afg09-2990 and Kosovo Hoti did not demonstrate clear differences in clinical outcome attributable to viral replication kinetics alone [212]. Together, these data suggest that host inflammatory responses may play a greater role in determining disease severity than viral replication dynamics alone.

Strain-specific differences in target cell susceptibility may also contribute to pathogenic heterogeneity. Mouse macrophages are more permissive to infection with IbAr10200 than with Turkey-2004, corresponding with increased lethality in the transiently immunosuppressed mouse model [119], although this difference is not observed in the more stringent intraperitoneal IFNAR^−/−^ mouse model [231]. Similarly, human monocyte-derived macrophages appear more permissive to infection with CCHFV strains associated with higher case fatality rates in humans [233]. These observations suggest that variation in macrophage susceptibility may influence pathogenic potential, although the impact of this phenotype likely depends on host species, immune status, and route of infection.

### Immune dysregulation and hyperinflammation

CCHFV disease is consistently associated with a dysregulated inflammatory response that contributes to vascular dysfunction, coagulopathy, and organ failure. The immunobiology of CCHFV has been comprehensively reviewed by Rodriguez, Hawman et al. [175]. Mechanistically, infected macrophages and DCs produce a broad array of cytokines and chemokines while supporting viral replication. Cytokines released from infected DCs, particularly TNF-α, can activate endothelial cells and thereby link immune activation directly to vascular pathology [171–173,234]. Clinical markers supporting the presence of significant inflammatory responses in severe disease include high-sensitivity C-reactive protein (hsCRP) and plasma fetuin-A (PFA). HsCRP is a positive acute-phase protein that has been found to be severely (>10-fold) elevated in CCHFV patients [235]. PFA, a negative acute-phase protein that normally decreases during inflammation to avoid suppressing antiviral immune responses, has been reported to be significantly lower in fatal CCHF cases compared with survivors [236]. A similar pattern has been observed in sepsis, where PFA levels are significantly reduced in non-surviving patients with septic shock compared with survivors [237]. Although PFA has both pro- and anti-inflammatory properties, experimental studies indicate that it can confer protection in lethal models of sepsis, potentially through modulation of innate immune responses and suppression of excessive macrophage activation [238–240]. The exact role of PFA in CCHFV pathogenesis is unclear.

Clinical and experimental studies indicate that this inflammatory response is characterized primarily by elevated cytokine signaling rather than lipid mediator-driven inflammation. For example, reduced 5-lipoxygenase (5-LO) activity and lower arachidonic acid levels have been observed in CCHF patients compared with healthy controls, while cysteinyl leukotriene levels remain comparable overall but are significantly increased in fatal cases relative to survivors [241,242]. Notably, thromboxane A_2_, a downstream product of arachidonic acid via the cyclooxygenase pathway, was not measured in these studies. As a result, it remains unclear whether the reduced arachidonic acid and 5-LO reflect substrate diversion or more general attenuation of eicosanoid biosynthesis.

#### Cytokine dysregulation

Across clinical cohorts and experimental systems, severe CCHF is consistently associated with elevated pro-inflammatory cytokines and chemokines. Increased concentrations of TNF-α, IL-8, IL-9, IL-15, IP-10, MCP-1, and IL-36α have been repeatedly correlated with disease severity and poor outcomes [95,176,207,209,232,243–245]. Among these mediators, TNF-α appears to play a particularly important role in disease progression. In a transiently immunosuppressed mouse model of CCHF, treatment with anti-TNF-α monoclonal antibodies conferred approximately 50% protection from lethal disease, supporting a pathogenic role for excessive inflammatory signaling [232]. While some cytokines, such as IL-12 and IL-13, remain relatively unchanged across species and experimental systems, others demonstrate context-dependent associations with severity. For example, IL-6 levels vary across patient cohorts and experimental models, suggesting that its role may depend on disease stage or host background [95,244,246].

Mechanistic insight into the drivers of cytokine dysregulation has emerged from studies examining MAVS, a key adaptor in RIG-I-mediated innate immune signaling. In mice infected with the lethal strain Afg09-2990, MAVS deficiency conferred protection from weight loss, clinical disease, and mortality, accompanied by markedly reduced concentrations of TNF-α, IL-6, IFN-γ, IL-18, CCL2, and CCL7 compared with wild-type animals [232]. Notably, loss of MAVS, but not melanoma differentiation-associated protein 5 (MDA5), improved survival even when type I IFN signaling was absent, suggesting that MAVS-dependent inflammatory signaling contributes directly to disease pathogenesis rather than antiviral protection alone [232].

Anti-inflammatory pathways appear less well characterized but also contribute to immune dysregulation. Elevated IL-10 concentrations are frequently observed in severe or fatal cases and may promote immune suppression and enhanced viral replication, a pattern similarly observed in lethal mouse models [95,176,207,243]. In addition to altered cytokine levels, regulatory mechanisms that normally restrain inflammatory signaling appear disrupted. Transcriptomic and microRNA profiling studies in patients demonstrate widespread dysregulation of cytokine regulatory networks, including altered expression of miRNAs predicted to target key inflammatory mediators such as IL-1 receptor-associated kinase-1 binding protein-1 (IRAK1BP1) and multiple IL signaling pathways, including IL-6, IL-12, and IL-2 receptor signaling [190,192]. Downregulation of miR-18a-5p, which normally targets suppressor of cytokine signaling-5, has also been reported in CCHF patients, suggesting that loss of this regulatory axis may further amplify inflammatory signaling [190].

Although many of these observations remain correlative and vary between cohorts and model systems, converging evidence from clinical, experimental, and transcriptomic studies supports a model in which dysregulated host inflammatory responses, rather than direct viral cytopathology alone, play a major role in driving disease progression.

#### Monocyte/Macrophage activation, hemophagocytosis, and T-cell dysfunction

Severe CCHF is also characterized by pronounced monocyte-macrophage activation, with features resembling hyperinflammatory syndromes such as MAS)and HLH-like disease (Figure 3). Clinical studies demonstrate elevated levels of biomarkers associated with systemic monocyte and macrophage activation, including neopterin, ferritin, and presepsin in patients with severe or fatal disease (Table 1) [241,247–252]. Other markers such as myeloperoxidase (MPO) are also elevated in patients [248,253]. Chitotriosidase, an enzyme produced by activated macrophages, was found to be significantly decreased in fatal patients but elevated in CCHF patients overall, possibly reflecting immune exhaustion, leukopenia, or dysregulated macrophage responses during later stages of disease [254]. Additional indicators of macrophage-driven hyperinflammation include elevated IL-6 and transforming growth factor-β1 (TGF-β1), along with declining platelet-derived growth factor subunit B (PDGF-B), all of which correlate with disease severity and mortality [117,241,255]. Experimental models further support a role for macrophage activation in CCHFV pathogenesis. In the transiently immunosuppressed mouse model, infection with the uniformly lethal IbAr10200 strain resulted in significantly elevated plasma CD163 concentrations compared with animals infected with less virulent strains, consistent with macrophage activation and hemophagocytic pathology [119].

**Table 1.** Summary of macrophage activation and T cell regulatory biomarkers in CCHFV infection by disease severity and clinical outcome.

Analyte	Total patients	Mild	Moderate	Severe	Survivor	Non-survivor	Reference
Neopterin	↑↑↑↑*	NR	NR	NR	↑↑↑↑	↑↑↑↑↑^+^	[252]
Ferritin^#^	↑↑*	↑^a^	↑↑^ab^	↑↑↑^c^	↑↑	↑↑↑↑^+^	[241,251]
Presepsin	↑↑*	↑^a^	↑↑^b^	↑↑↑^c^	NR	NR	[249]
Plasma MPO	↑*	NR	NR	NR	NR	NR	[248]
Leukocyte MPO	↑*	NR	NR	NR	NR	NR	[248]
Chitotriosidase	↑*	NR	NR	NR	↑	↓↓↓*^+^	[254]
TGF-β1	↑*	NR	↑^a^	↑^b^	↑	↑	[255]
TGF-β	↓*	NR	↓^a^	↓^b^	NR	NR	[258]
Phosphorylated Foxp3	↓*	NR	NR	NR	NR	NR	[259]

Analyte fold changes are expressed relative to healthy controls or reference ranges. Arrows indicate the direction and magnitude of change: ↑ or ↓, >1.5–2-fold; ↑↑, >3-fold; ↑↑↑, >4-fold; ↑↑↑↑, >10-fold; ↑↑↑↑↑, >30-fold. (#) indicates studies involving pediatric populations. (*) indicates a statistically significant difference compared with the study control group. (a), (b), and (c) denote statistically significant differences between severity cohorts. (+) indicates a statistically significant difference between survivor and non-survivor cohorts. NR, not reported; MPO, myeloperoxidase. Statistical significance was defined as *p* < 0.05.

In parallel with macrophage activation, alterations in T-cell regulation have also been reported. Elevated soluble IL-2 receptor (sIL-2 R), a marker of T-cell activation, correlates with disease severity in pediatric CCHF cases and may be associated with HLH-like clinical features [256] and MAS-like clinical features [257]. TGF-β1, which exerts immunoregulatory effects by inhibiting T-cell proliferation and IL-2 production, has been reported as both upregulated and downregulated across studies [255,258]. These paradoxical findings may reflect dysregulation of regulatory T cells (Tregs). In support of this, reduced phosphorylation of the transcription factor Foxp3, which is critical for Treg suppressive function, was identified in CCHF patients. Impaired Treg activity may contribute to inadequate immune regulation, excessive inflammation, and progression to severe disease [259].

The sustained inflammatory environment generated by activated monocytes, macrophages, and dysregulated T-cell responses likely contributes to downstream cellular stress pathways, including mitochondrial dysfunction, oxidative stress, and programmed cell death observed in infected tissues.

### Cellular stress and death pathways

#### Intracellular stress and cell-death programs

**Figure 5.**
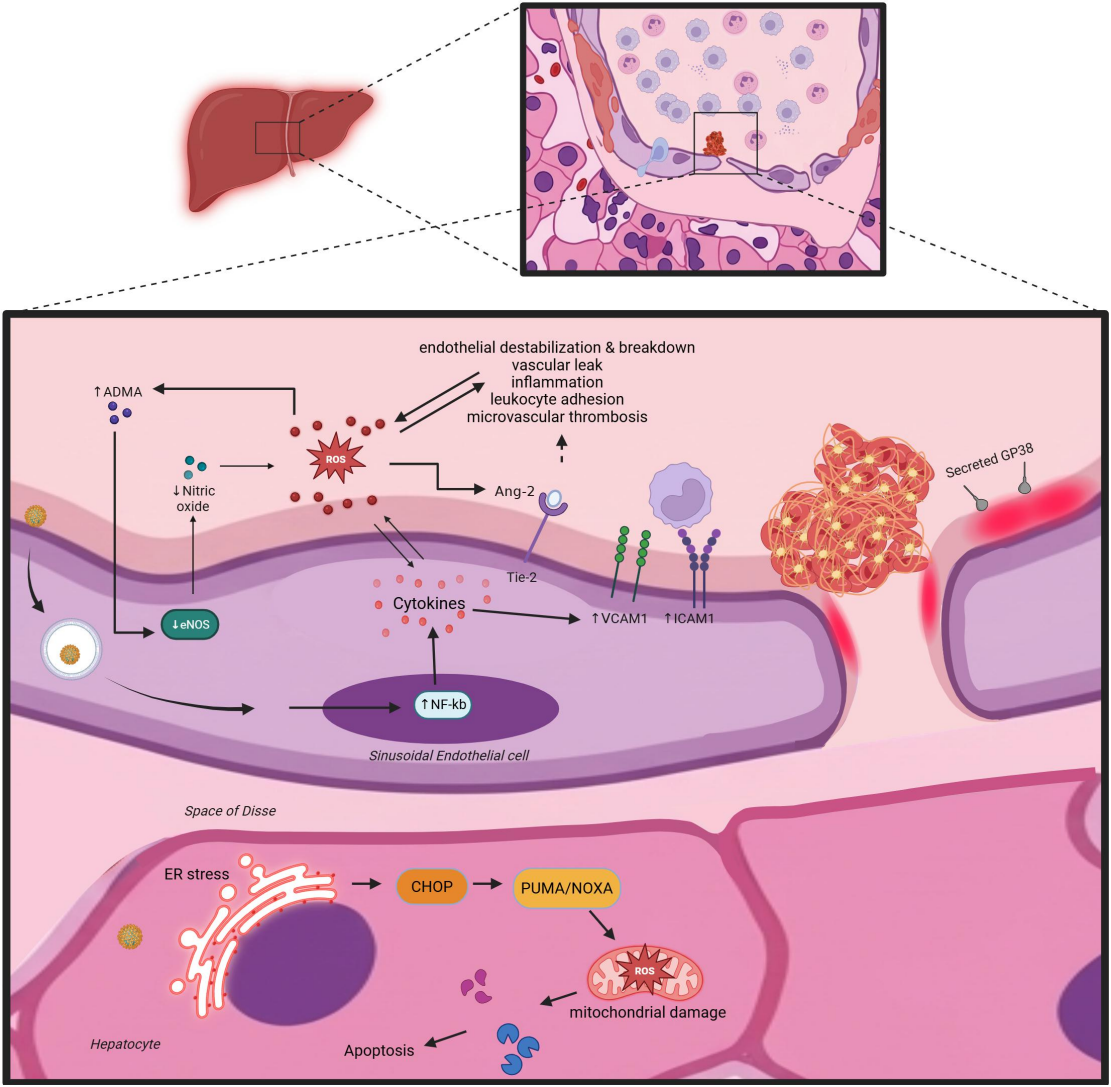
Apoptosis and endothelial dysfunction. The liver is a major target organ of CCHFV. Top right inset: liver sinusoids demonstrating intravascular Kupffer cell hyperplasia, monocyte and lymphocyte infiltration, and microthrombus formation. Bottom inset: CCHFV induced intracellular stress and death pathways and endothelial dysfunction. Both production of reactive oxygen species (ROS) from the pro-inflammatory response and activation of NF-kB signaling facilitate a progressive cascade leading to endothelial compromise, vascular leak, and microvascular thrombosis. The ROS production leads to an increase in accumulation of asymmetric dimethylarginine (ADMA) which results in endothelial nitric oxide synthase (eNOS) inhibition and nitric oxide (NO) depletion. NO depletion results in continued production of ROS. Cytokine exposure and ROS cause endothelial cells to rapidly release angiopoietin-2 (Ang-2) which antagonizes Tie-2 resulting in loss of endothelial quiescence and destabilization of endothelial junctions and increased sensitivity to inflammatory cytokines, driving vasoconstriction and microvascular dysfunction. These activated endothelial cells express more VCAM-1 and ICAM-1 leading to leukocyte adhesion and thrombus formation. GP38 has a toxic effect on the glycocalyx. Inflammatory and viral-induced ER stress induce CHOP and activation of the PUMA/NOX pathway leading to mitochondrial damage, the production of ROS, cytochrome C release, and caspase activation which result in cellular apoptosis.

CCHFV infection triggers multiple cellular stress responses that converge on apoptotic and inflammatory injury with differentially regulated apoptotic pathways (Figure 5). Early during infection, the virus modulates apoptosis to counter host antiviral defenses. The viral NP suppresses intrinsic apoptotic signaling by inhibiting caspase-3 and caspase-9 activation and blocking Bcl-2–associated X protein-mediated cytochrome-c release, while the NSs protein disrupts mitochondrial membrane potential [149,260]. As infection progresses and viral replication increases, cellular stress responses become more pronounced. In hepatocytes, CCHFV infection is associated with ER stress responses and upregulation of pro-apoptotic factors such as the p53-upregulated modulator of apoptosis (PUMA)/NOXA axis [261]. Consistent with these mechanisms, circulating markers of apoptosis including increases in perforin, caspase-3, and decreases in soluble Fas ligand are observed in infected patients (Table 2) [251]. These trends suggest a relative shift in circulating apoptotic signaling toward cytotoxic granule-mediated mechanisms, although these markers do not fully resolve cell-type-specific pathway activity.

**Table 2.** Apoptosis and oxidative stress biomarkers in CCHFV infection stratified by disease severity.

Analyte	Total patients	Mild	Moderate	Severe	Reference
Perforin^#^	NR	NR	↑↑*	↑↑*	[251]
Caspase-3^#^	NR	NR	↑*	↑*	[251]
Fas ligand^#^	NR	NR	↓*	↓*	[251]
LPO^#^	↑*	NR	NR	NR	[263]
Erythrocyte MDA	↑*	NR	NR	NR	[266]
Plasma MDA	↑*	NR	NR	NR	[266]
IMA	↑*	NR	↑	↑*	[267]
NOX-1	↑*	↑*	↑*	NR	[268]
NOX-2	NSD	NSD	NSD	NR	[268]
NOX-4	NSD	NSD	NSD	NR	[268]
NOX-5	↑↑↑↑↑*	↑↑↑↑↑*	↑↑↑↑^*+^^	NR	[268]
PON^#^	↓*	NR	NR	NR	[253,266]
Arylesterase^#^	↓*	NR	NR	NR	[253,266]
Catalase U/L	NSD, ↓↓*	NR	NR	NR	[253,266]
Vitamin E	↓*	NR	NR	NR	[266]
Total thiol^#^	↓ - ↓↓↓*	NR	NR	NR	[253,264,265]
SOD	↑*	NR	NR	NR	[266]
Ceruloplasmin	↑*	NR	NR	NR	[253]

Analyte fold changes are expressed relative to healthy controls or reference ranges. Arrows indicate the direction and magnitude of change: ↑ or ↓, >1-fold; ↑↑ or ↓↓, >2-fold; ↑↑↑ or ↓↓↓, >5-fold; ↑↑↑↑, >8-fold; ↑↑↑↑↑, >9-fold. (#) indicates studies involving pediatric populations. (*) indicates a statistically significant difference compared with the study control group. (+) indicates a statistically significant difference between survivor and non-survivor cohorts. (^) indicates values statistically lower than those observed in mild disease. NSD, no significant difference detected; NR, not reported; LPO, lipid peroxidation; MDA, malondialdehyde; IMA, ischemia-modified albumin; NOX, NADPH oxidase; PON, paraoxonase; SOD, superoxide dismutase. Statistical significance was defined as *p* < 0.05.

Viral replication also induces significant ER stress. Markers associated with all three branches of the unfolded protein response are upregulated, indicating that viral protein production overwhelms ER folding capacity and contributes to apoptosis and inflammatory injury [214]. Gene-expression analyses from patients with severe or fatal disease further suggest involvement of extrinsic apoptotic pathways, where inflammatory cytokines suppress NF-κB-mediated survival signaling and sensitize cells to TNF-α–driven apoptosis [262].

#### Systemic oxidative stress and redox imbalance

Intracellular stress responses during infection intersect with broader disturbances in oxidative balance that contribute to cellular injury and tissue damage (Figure 5, bottom inset). Viral replication and inflammatory signaling promote mitochondrial dysfunction and generation of reactive oxygen species (ROS), potentially involving ER stress-associated pathways (e.g. CHOP) and upregulation of pro-apoptotic factors such as PUMA/NOXA-linked pathways [261]. These processes contribute to endothelial injury and systemic oxidative stress. Consistent with this, patients exhibit elevated markers of oxidative damage, including lipid peroxides, malondialdehyde (MDA), and ischemia-modified albumin (IMA), alongside reduced antioxidant defenses such as paraoxonase, arylesterase, catalase, vitamin E, and total thiols (Table 2) [253,263–267]. Elevated expression of NADPH oxidase enzymes NOX-1 and NOX-5, which generate reactive oxygen species, provides additional evidence of increased oxidative stress during infection. Interestingly, reduced NOX-5 levels have been associated with more severe disease, thrombocytopenia, and coagulation abnormalities, indicating a potentially complex role for NOX-derived ROS in disease progression [268]. NOX-derived ROS may play a dual role by contributing to oxidative injury while also supporting antiviral or hemostatic responses, with loss of NOX-5 potentially reflecting dysregulated host defense in severe disease.

Though many antioxidant analytes are measurably low in patients, some antioxidant analytes are increased. Upregulation of the IFN-inducible metallothionein gene MT1L has been observed in CCHF patients [190]. Metallothioneins act as metal-binding antioxidants that buffer reactive oxygen species and inflammatory metal fluxes, suggesting that MT1L induction may represent a compensatory response to oxidative stress. Likewise, ceruloplasmin, a copper-carrying protein that plays a role in metabolism and antioxidant defense, was elevated in CCHF patients [253]. Superoxide dismutase, an antioxidant enzyme that neutralizes superoxide radicals, has also been observed to be elevated in patients [266]. It remains unclear whether these responses represent compensatory mechanisms to restore redox balance or acute-phase inflammatory responses. These stress responses also disrupt cellular energy homeostasis, highlighting the close coupling between oxidative stress, mitochondrial dysfunction, and metabolic reprogramming during CCHFV infection.

### Metabolic and mitochondrial reprogramming

#### Metabolic alterations

CCHFV infection induces substantial metabolic remodeling involving both lipid and AA metabolism. Metabolomic studies in CCHF patients demonstrate alterations in pathways related to biosynthesis of unsaturated fatty acids, α-linolenic acid metabolism, arachidonic acid metabolism, fatty acid biosynthesis, aminoacyl-tRNA biosynthesis, arginine biosynthesis, nitrogen metabolism, and glutamine-glutamate metabolism [242,269,270]. These findings suggest that infection reshapes central metabolic pathways that support both host immune responses and viral replication.

Lipidomic analyses reveal pronounced perturbations in fatty acid composition during infection. Patients with severe or fatal disease exhibit higher omega-6/omega-3 and linoleic acid/dihomo-γ-linolenic acid ratios compared with patients with milder disease [242]. In contrast, concentrations of several very-long-chain fatty acids, including arachidonic acid, behenic acid, lignoceric acid, and cerotic acid, are reduced in severe case groups (Table 3) [242]. These lipid shifts may reflect altered inflammatory lipid mediator production and metabolic reprogramming associated with disease progression.

**Table 3.** Lipid and amino acid metabolic biomarkers in CCHFV infection stratified by disease severity and outcome.

Analyte	Total patient	Mild	Moderate	Severe	Survivor	Non-survivor	Reference
LA/DGLA ratio	↑↑*	↑^a^	↑↑↑^b^	↑↑↑↑^b^	NR	NR	[242]
Arachidonic acid	↓*	↓	↓	↓	NR	NR	[242]
α-aminobutyric acid	↑↑*	↑↑^a^	↑↑^b^	↑↑^b^	NR	NR	[269]
GABA	↑*	NR	NR	NR	↑	↑^+^	[270]
Glutamic acid	↑*	↑ ^a^	↑↑^b^	↑↑↑^c^	NR	NR	[269]
Glutamate	↑↑*	NR	NR	NR	↑↑	↑↑^+^ *(statistically lower than survivors)*	[270]
Phenylalanine	↑*	↑^a^	↑^b^	↑↑↑↑^c^	NR	NR	[269]
Aspartic acid	↑↑↑↑*	NR	NR	NR	↑↑*	↑↑↑↑^+^	[270]
Glutamine	↓*	↓^a^	↓^a^	↓↓^b^	↓	↓	[269,270]
Alanine	↓*	↓^a^	↑^b^	↑↑^c^	NR	NR	[269]
Ethanolamine	↓*	↓^a^	↑^b^	↑↑^c^	NR	NR	[269]
Citrulline	↓*	↓^a^	↓^a^	↓^b^	NR	NR	[269]
Taurine	↓↓↓*	↓↓↓^a^	↓↓↓↓^b^	↓↓^a^	NR	NR	[269]
Arginine	↓*	↓^a^	↓↓↓^b^	↓↓↓↓↓^c^	NR	NR	[269]

Analyte fold changes are expressed relative to healthy controls or reference ranges. Arrows indicate the direction and magnitude of change: ↑ or ↓, >1-fold; ↑↑ or ↓↓, >2-fold; ↑↑↑ or ↓↓↓, >3-fold; ↑↑↑↑ or ↓↓↓↓, >4-fold; ↓↓↓↓↓, >30-fold decrease. (*) indicates a statistically significant difference compared with the study control group. (+) indicates a statistically significant difference between survivor and non-survivor cohorts. NR, not reported; LA/DGLA ratio, linoleic acid/dihomo-γ-linolenic acid ratio; GABA, gamma-aminobutyric acid. Statistical significance was defined as *p* < 0.05.

Amino acid metabolism is also significantly altered during infection. Severe CCHF is associated with increased concentrations of α-aminobutyric acid, gamma-aminobutyric acid (GABA), glutamic acid, glutamate, phenylalanine, and aspartic acid, alongside reduced levels of glutamine, alanine, arginine, β-alanine, ethanolamine, citrulline, and taurine (Table 3) [269,270]. Glutamic acid and phenylalanine increase progressively with disease severity and are particularly elevated during the hemorrhagic phase [269]. In contrast, arginine levels decline with increasing disease severity. Arginine depletion may reflect the increase in arginase activity from myeloid cells, disruption of the hepatic urea cycle from liver dysfunction, as well as type I IFN-mediated suppression of arginiosuccinate synthase 1 in infected hepatocytes [269,271,272].

Because arginine, and indirectly citrulline, are required for nitric oxide production and effective CD8^+^ T-cell activation, reductions in these AAs may contribute to endothelial dysfunction, impaired antiviral immunity, and increased disease severity [273]. Ethanolamine is a precursor for the synthesis of phospholipids, thus a reduction in this AA may have broader lipid metabolism effects. Comparable metabolic alterations have been described in other viral infections, suggesting that these changes reflect shared host metabolic dependencies exploited during infection [274–277]. Together, these findings indicate that metabolic reprogramming occurs in parallel with cytokine dysregulation, immune activation, and oxidative stress, linking altered cellular metabolism to the tissue injury, coagulopathy, and vascular dysfunction observed in severe CCHF.

While these metabolomic signatures provide insight into systemic metabolic disturbances during infection, transcriptomic and proteomic studies indicate that these changes are accompanied by substantial mitochondrial and bioenergetic reprogramming within infected cells.

#### Mitochondrial and bioenergetic reprogramming

Transcriptomic and proteomic studies indicate that CCHFV infection drives substantial mitochondrial and metabolic remodeling. Early during infection, central carbon metabolism, including pyruvate utilization and tricarboxylic acid cycle activity, appears transiently suppressed, potentially favoring viral replication before partially recovering by approximately 48 hours post- infection [213,278]. This shift is accompanied by increased oxidative phosphorylation and fatty acid oxidation, suggesting a redistribution of cellular energy production toward mitochondrial respiration rather than glycolysis during acute infection [213,278].

Metabolic remodeling is closely linked to changes in host immune signaling pathways. Studies report concurrent upregulation of N-glycan biosynthesis and cytokine-receptor interaction pathways, together with downregulation of T-cell receptor signaling, T helper cell differentiation (Th1/Th2), and natural killer cell cytotoxicity pathways [213,278]. These alterations suggest that metabolic reprogramming during infection is tightly coupled to immune dysregulation and impaired antiviral effector responses.

Collectively, the redistribution of metabolic flux toward AA metabolism, oxidative phosphorylation, and lipid utilization appears to be a characteristic feature of acute CCHFV infection and may influence both viral replication efficiency and host immune function [278]. These findings support a model in which mitochondrial and metabolic reprogramming contribute to CCHF pathogenesis by linking cellular energetics with immune dysfunction and downstream tissue injury.

### Endothelial dysfunction and hemostatic failure

Like other viral hemorrhagic fevers, CCHF is characterized by profound disturbances in vascular integrity and hemostasis driven by complex interactions between viral replication and host immune responses that lead to endothelial injury and activation of coagulation pathways [279]. These resulting hemorrhagic manifestations reflect the convergence of endothelial barrier disruption, platelet consumption and dysfunction, and secondary coagulation abnormalities.

#### Endothelial dysfunction

Endothelial activation and injury are central features of CCHF pathogenesis and appear to arise predominantly from indirect inflammatory mechanisms rather than widespread direct viral infection of endothelial cells (Figure 5) [166,172]. Early *in vitro* studies demonstrated that supernatants from CCHFV-infected monocyte-derived dendritic cells (moDCs), and not CCHFV alone, robustly activated human endothelial cells implicating the soluble inflammatory mediators as the key drivers for endothelial dysfunction [172].

Clinical observations consistently support widespread endothelial perturbation in CCHF. Clinical markers of endothelial activation, injury, and dysfunction in CCHF patients include elevated levels of endothelin-1 (ET-1), endocan, c-type natriuretic peptide (CNP), and hyaluronic acid (Table 4) [256,280–282].

**Table 4.** Vascular and endothelial biomarkers in CCHFV infection stratified by disease severity and outcome.

Analyte	Total patients	Mild	Moderate	Severe	Survivor	Non-survivor	Reference
Pediatric ET-1	NR	NR	↑*^a^	↑*^b^	NR	NR	[256]
ET-1 (pg/mL)	↑↑*	NR	↑↑^a^	↑↑↑^b^	↑↑	↑↑↑^†^	[281,287]
Endocan	↓↓↓*	↓↓↓	↓↓↓	NR	NR	NR	[235]
CNP	NR	NR	↑*^a^	↑*^b^	NR	NR	[280]
Hyaluronic acid	↑↑↑^^^	NR	NR	NR	↑↑↑	↑↑↑^+^	[282]
Pediatric-HIF-1α	↑*	NR	NR	NR	NR	NR	[286]
sVCAM-1	↑*	NR	↑↑^a^	↑↑ ^b^	↑↑	↑↑^+^	[282,285]
Log sICAM-1	↓*	NR	↓^a^	↓^b^	↓	↓^+^	[282,285]
ICAM-1	↑*	NR	↑	↑	NR	NR	[287]
Pediatric– VEGF-A	↑*	NR	NR	NR	NR	NR	[286]
VEGF	↓*	NR	↓^a^	↓↓↓^b^	↓^c^	↓↓↓^+^	[285]
VEGF	↑*	NR	↑^a^	↑^b^	↑	↑^+^	[282,283]
sVEGF-R1	↑*	NR	↑^a^	↑↑^b^	↑	↑↑↑^+^	[283]
Log sE-selectin	↓*	NR	↓	↓	↓	↓	[285]
Log sP-selectin	↓*	NR	–	–	–	–	[285]
sL-selectin	↑*	NR	↑	↑	↑	↑	[285]
ADMA	↓*	NR	↓^a^	↑^b^	NR	NR	[287]
Pediatric Ang-2	↑*	NR	↑^a^	↑^b^	NR	NR	[284]
Ang-2	↓	NR	↓	↓^*b^	↓	↓↓^†^	[281]
Pediatric Tie-2	↑*	NR	↑^a^	↑^b^	NR	NR	[284]
Tie-2	↑↑↑*	NR	↑↑^a^	↑↑↑^*b^	↑↑↑	↑↑↑^†^	[281]

Analyte fold changes are expressed relative to healthy controls or reference ranges. Arrows indicate the direction and magnitude of change: ↑ or ↓, >1-fold; ↑↑ or ↓↓, >5-fold; ↑↑↑ or ↓↓↓, >10-fold. (*) indicates a statistically significant difference compared with the study control group. (+) indicates a statistically significant difference between survivor and non-survivor cohorts. (†) indicates sample size insufficient to determine statistical significance between severity groups. (–) indicates values reported in a source publication that were not included because they could not be confidently interpreted. NSD, no significant difference detected; NR, not reported; ET-1, endothelin-1; CNP, C-type natriuretic peptide; HIF-1α, hypoxia-inducible factor-1α; ADMA, asymmetric dimethylarginine. Statistical significance was defined as *p* < 0.05.

Dysregulation of angiogenic mediators and adhesion molecules is also a prominent feature. Increased levels of hypoxia inducible factor 1-α (HIF1-α), soluble vascular cell adhesion molecule-1 (sVCAM-1), soluble intercellular adhesion molecule-1 (sICAM-1), VEGF, and selectin family members have been observed, often differing between survivors and non-survivors (Table 4) [281–286]. Notably, these changes are not uniform. While sVCAM-1 is consistently elevated, soluble ICAM-1 and selectins (E-, P-, and L-selectin) demonstrate divergent patterns, suggesting selective rather than global endothelial activation. Reported differences in VEGF and adhesion molecule levels in severe disease likely reflect variation in disease stage, sampling time, or concurrent increases in soluble decoy receptors such as sVEGFR-1, rather than true biological contradiction [282,283,285].

Additionally, analytes further highlight disruption of endothelial regulatory pathways. Asymmetric dimethylarginine (ADMA), an endogenous inhibitor of nitric oxide synthase, decreases in patients overall but increases significantly in severe disease. This pattern may reflect a compensatory mechanism to preserve nitric oxide bioavailability in non-severe cases and a nitric oxide impairment and failure in the late phase or severe cases [256,287].

Alterations in angiopoietin-Tie-2 axis show distinct age- and severity-dependent patterns. In pediatric patients, both Ang-2 and Tie-2 levels were significantly elevated in severe disease, consistent with impaired endothelium stability [284]. In contrast, adult patients showed significantly lower Ang-2 levels, with more pronounced decreases in fatal cases, alongside increased circulating Tie-2. This imbalance may be due to depleted reserves of Ang-2 within Weibel-Palade bodies, increased VEGF-mediated shedding of the Tie-2, or a result of increased circulating decoy receptor activity.

Extracellular matrix remodeling is also dysregulated during CCHFV infection (Table 5). Platelet-derived growth factor (PDGF-B), which supports vascular integrity, was significantly decreased in patients [255]. Matrix metalloproteinases (MMP-1, −2, −7, −9, and −10) are elevated in patients, while their inhibitor TIMP-1 is reduced in severe disease, consistent with destabilization of the vascular basement membrane and increased permeability [288]. Ghrelin, a peptide with vasoprotective and anti-inflammatory properties that can suppress ET-1 signaling, is significantly decreased in severe disease, further contributing to endothelial dysfunction and dysregulated vascular tone [289].

**Table 5.** Tissue remodeling biomarkers in CCHFV infection stratified by disease severity and outcome.

Analytes	Total patients	Moderate	Severe	Survivor	Non-survivor	Reference
PDGF-B	↓*	↓^a^	↓^b^	↓	↓	[255]
MMP-1	↑*	NR	NR	↑* *(statistically lower than acute disease)*	N/A	[288]
MMP-2	↑*	NR	NR	NSD	N/A	[288]
MMP-7	↑*	NR	NR	↑* *(statistically lower than acute disease)*	N/A	[288]
MMP-9	↑*	NR	NR	↑* *(statistically lower than acute disease)*	N/A	[288]
MMP-10	↑*	NR	NR	↑* *(statistically lower than acute disease)*	N/A	[288]
(TIMP-1)	↑*	↑*	↑* *(statistically lower than moderate disease)*	↑* *(statistically lower than acute disease)*	N/A	[288]
Ghrelin	↓↓*	↓^a^	↓↓↓^b^	NR	NR	[289]

Analyte fold changes are expressed relative to healthy controls or reference ranges. (^) indicates comparisons made against published reference ranges rather than an internal study control. Arrows indicate the direction and magnitude of change: ↑ or ↓, >1-fold; ↑↑ or ↓↓, >5-fold; ↑↑↑ or ↓↓↓, >10-fold; ↑↑↑↑ or ↓↓↓↓, >30-fold. (*) indicates a statistically significant difference compared with the study control group. (+) indicates a statistically significant difference between survivor and non-survivor cohorts. (†) indicates sample size insufficient to determine statistical significance between severity groups. NSD, no significant difference detected; NR, not reported; PDGF-B, platelet-derived growth factor-B; MMP, matrix metalloproteinase; TIMP-1, tissue inhibitor of metalloproteinases-1. Statistical significance was defined as *p* < 0.05.

Although endothelial dysfunction is primarily attributed to host inflammatory responses, recent evidence suggests that viral factors may also contribute directly. A recent study demonstrated that the secreted CCHFV glycoprotein GP38 acts as a viral toxin capable of inducing endothelial barrier dysfunction independently of viral replication. GP38 disrupts the endothelial glycocalyx, induces endothelial hyperpermeability, and promotes vascular leak *in vivo*, particularly in the liver and spleen [290].

Disruption of endothelial barrier integrity and glycocalyx structure also promotes platelet adhesion, activation, and consumption at sites of vascular injury. These processes link endothelial dysfunction directly to the thrombocytopenia and coagulopathy that characterize severe CCHF.

#### Coagulopathies

Coagulopathy is a central feature of CCHFV infection and reflects a complex interplay between endothelial dysfunction, dysregulated coagulation, and altered fibrinolysis. The hemostatic disturbance in CCHF is best understood as a dynamic imbalance between clot formation, consumption of hemostatic components, and impaired clot stability.

Endothelial injury serves as a primary initiating event. Activation and damage of the endothelium promote the release of procoagulant mediators, such as von Willebrand factor (vWF), which has been shown to be elevated in CCHFV patients [287]. Thrombomodulin levels appear to vary with disease severity, with reduced levels in moderate disease and elevated circulating levels in severe disease (Table 6) [287]. This shift favors a procoagulant state with reduced regulatory control, leading to widespread activation of the coagulation cascade. Clinical and laboratory findings support a model of consumptive coagulopathy. Prolongation of prothrombin time (PT) and activated partial thromboplastin time (aPTT), together with reduced fibrinogen levels, reflect depletion of coagulation factors secondary to ongoing thrombin generation. Elevated D-dimer further indicates active fibrin formation and degradation, suggesting that clot formation is occurring but is not effectively stabilized. Collectively, these findings are consistent with a consumptive, DIC-like process, particularly in severe and fatal disease [247].

**Table 6.** Coagulation and fibrinolysis biomarkers in CCHFV infection stratified by disease severity and outcome.

Analyte	Total patients	Moderate	Severe	Survivor	Non-survivor	Reference
vWF	↑↑*	↑↑*	↑↑*	NR	NR	[287]
TM	↑*	↓^a^	↑^b^	NR	NR	[287]
TAFI	↓↓↓*	↓↓↓	↓↓↓	↓↓	↓↓	[291]
TAFI activity	↓*	NR	NR	NR	NR	[292]
tPA	NR	NR	NR	*WNL*^*^*^	↑^+^, *WNL*^*^*^	[293]
PAI-1	NR	NR	NR	*WNL*^*^*^	↑↑^+^, *WNL*^^^	[293]
IPF	↑↑*	NR	NR	NR	NR	[294]

Analyte fold changes are expressed relative to healthy controls or reference ranges. (^) indicates comparisons made against published reference ranges rather than an internal study control. Arrows indicate the direction and magnitude of change: ↑ or ↓, >1-fold; ↑↑ or ↓↓, >2-fold; ↑↑↑ or ↓↓↓, >5-fold. (*) indicates a statistically significant difference compared with the study control group. (+) indicates a statistically significant difference between survivor and non-survivor cohorts. NSD, no significant difference detected; NR, not reported; TM, thrombomodulin; vWF, von Willebrand factor; IPF, immature platelet fraction; TAFI, thrombin-activatable fibrinolysis inhibitor; PAI-1, plasminogen activator inhibitor-1; tPA, tissue plasminogen activator; WNL, within normal limits. Statistical significance was defined as *p* < 0.05.

Fibrinolytic pathways are also dysregulated and contribute to clot instability. Under physiological conditions, thrombin bound to thrombomodulin activates thrombin-activatable fibrinolysis inhibitor (TAFI), which stabilizes fibrin by limiting plasmin-mediated degradation. In CCHFV infection, reduced thrombomodulin expression, together with decreased TAFI levels and activity, suggests disruption of this regulatory axis [287,291,292]. As a result, fibrin clots may be structurally unstable and more susceptible to breakdown. This is further supported by alterations in fibrinolytic mediators. Tissue plasminogen activator (tPA) levels are increased, while plasminogen activator inhibitor-1 (PAI-1) is also elevated but appears insufficient to fully suppress fibrinolysis [293]. This imbalance results in a state of dysregulated fibrinolysis, in which fibrin degradation proceeds despite ongoing coagulation. The combination of elevated D-dimer, reduced fibrinogen, and impaired TAFI activity supports a model of simultaneous clot formation and excessive clot breakdown, rather than isolated hyperfibrinolysis or purely prothrombotic DIC. Transcriptomic and biomarker studies further support disruption of fibrinolytic regulation. Upregulation of *SERPINE1*, encoding PAI-1, has been observed in infected hepatocyte models, and elevated circulating PAI-1 levels correlate with disease severity in patients [191,200,285,293]. However, the magnitude of PAI-1 elevation may be insufficient to counterbalance increased fibrinolytic activity, reinforcing the concept that CCHF coagulopathy reflects a maladaptive equilibrium between coagulation activation and fibrinolysis.

Platelets play a central role within this dysregulated hemostatic environment, functioning both as mediators of hemostasis and as immune-responsive cells. Thrombocytopenia in CCHF appears to be driven primarily by peripheral consumption rather than bone marrow suppression. Increased immature platelet fraction (IPF), mean platelet volume (MPV), and platelet-large cell ratio indicate a compensatory response characterized by release of immature platelets during periods of increased demand [294]. Variability in platelet distribution width (PDW) across patient cohorts likely reflects different phases of disease rather than contradictory findings. Elevated PDW early during hospitalization in hemorrhagic patients suggests increased heterogeneity in platelet size due to accelerated peripheral consumption, release of immature platelets, and platelet activation in response to endothelial injury and inflammatory signaling [295]. In contrast, reduced PDW observed at the nadir of thrombocytopenia may reflect exhaustion of compensatory platelet production or sustained peripheral destruction overwhelming megakaryopoiesis [296]. Platelet consumption occurs in parallel with ongoing coagulation activation and fibrin turnover, linking thrombocytopenia directly to the consumptive coagulopathy described above. In this context, platelets are continuously recruited to sites of endothelial injury and incorporated into unstable thrombi, contributing to both microvascular obstruction and progressive depletion of circulating platelets.

Experimental models provide additional mechanistic insight into platelet involvement in CCHFV pathogenesis. In immunocompetent C57BL/6J mice infected with CCHFV, thrombocytopenia was accompanied by elevated plasma thrombopoietin levels and transcriptomic evidence of platelet functional reprogramming, consistent with increased platelet demand [297]. Notably, IFITM3 was markedly upregulated in platelets from infected mice, implicating IFN-mediated antiviral responses in platelet biology during infection. Mechanistic studies in human megakaryoblast (MEG-01) cells demonstrated that IFITM3 acts as a restriction factor against CCHFV by limiting viral entry through interactions with viral glycoproteins. Overexpression of IFITM3 reduced viral replication, whereas IFITM3 knockout enhanced infectivity, establishing a direct antiviral role for this IFN-stimulated gene in platelet progenitors [297]. Similar IFITM3 upregulation has also been observed in patients and nonhuman primates during acute and mild disease states [212,213].

## Host determinants of disease

The extent to which host genetic variation and immune responses contribute to morbidity and mortality in CCHF remains incompletely understood. Mechanistic investigation has historically been limited by the requirement for type I IFN-deficient mice to model disease. However, studies using genetically diverse Collaborative Cross mouse strains infected with mouse-adapted CCHFV demonstrate that host genetic background strongly influences disease outcome and can reproduce the full spectrum of clinical phenotypes observed in humans, ranging from asymptomatic infection to uniform lethality [298]. Some strains develop fatal disease with marked cytokine responses, whereas others succumb without significant cytokine elevation, and still others remain clinically asymptomatic despite viral replication. These findings mirror observations in human infection and suggest that disease severity reflects host-specific immune and inflammatory responses rather than viral burden alone.

### Demographic and physiologic modifiers of disease

Age and physiologic state influence clinical outcomes in CCHF. Pediatric cases generally have milder and shorter disease courses than adults, although severe pediatric infections have been reported [115,116,126,299]. This difference may reflect age-related inflammatory responses. Children exhibit a more restricted chemokine profile, with elevations largely limited to CCL4 and granulocyte colony-stimulating factor, whereas adults demonstrate broader and sustained chemokine activation [300]. Clinical laboratory profiles also differ between pediatric and adult patients. Children tend to have higher serum albumin and amylase levels and lower creatinine, creatine kinase, and hepatic transaminases. They also frequently display higher leukocyte counts, prolonged prothrombin times, and lower fibrinogen levels compared with adults [301].

Pregnancy is another physiologic condition associated with increased disease severity. CCHF in pregnant women is linked to higher maternal mortality and frequent fetal or neonatal loss through spontaneous abortion or maternal death [181]. Although the mechanisms remain unclear, gestational immune tolerance, increased vascular permeability, and the procoagulant yet consumptive hemostatic state of pregnancy may exacerbate disease progression [302].

### Genetic variation in innate immune and IFN signaling

Genetic variation in innate immune sensing pathways has been associated with susceptibility to CCHFV infection and disease severity. Polymorphisms in endosomal TLRs involved in viral nucleic acid recognition appear particularly relevant. TLR7, which signals through MyD88 to activate IFN regulatory factor 7 (IRF7) and NF-κB, has been linked to clinical outcomes in CCHF. In a cohort of 149 patients and 171 controls, TLR7 polymorphisms, including c.4-151A/G and IVS1 + 1817 G/T variants, were associated with susceptibility, severe disease, and mortality. The c.4-151GG genotype conferred increased risk, and specific haplotypes differed significantly between cases and controls [205]. Variants in TLR8 (Met1Val and −129C/G) and TLR9 (−1486T/C) also occurred more frequently in CCHF patients than controls, and the TLR8 −129 G/G and TLR9 − 1486C/C genotypes were enriched among severe or fatal cases [203]. Similarly, polymorphisms in TLR10, an immunomodulatory receptor, were linked to disease outcomes. The TLR10 720A/C and 992T/A variants were associated with susceptibility and fatal outcomes, with the 992AA genotype increasing risk and the 720CC genotype potentially conferring protection [303].

Variation in type I IFN genes may further influence susceptibility. In a Turkish cohort, the IFNA1 − 1823 (rs1332190) GG genotype was significantly more common in healthy controls than in CCHF patients, suggesting a protective effect. Conversely, the IFNA17 Ile184Arg (rs9298814) TG genotype occurred more frequently in infected individuals [304]. Although these variants were not associated with mortality, they indicate that host genetic differences affecting IFN signaling may influence infection susceptibility.

### Genetic and epitranscriptomic regulation of inflammatory signaling

Variation in intracellular inflammatory signaling pathways may also influence host responses to CCHFV infection. The NF-κB pathway, a central regulator of antiviral and inflammatory gene expression, has been implicated in disease susceptibility and outcome. The NF-κB1 − 94 D allele (WD and DD genotypes) occurs more frequently in CCHF patients than in controls, and individuals carrying the DD genotype have an increased risk of infection. Notably, 71.4% of fatal cases carried this allele, and the DD genotype was associated with a fourfold higher risk of fatal disease [305]. This polymorphism is linked to reduced NF-κB1 transcriptional activity, suggesting altered inflammatory gene regulation may influence disease progression [306]. Polymorphisms in NF-κBIA, which encodes the inhibitory protein IκBα, have also been associated with increased disease risk. Individuals carrying the 3′UTR G allele have approximately a twofold greater risk of infection, and this variant was present in 85.7% of fatal cases [305].

Epitranscriptomic regulation may represent another layer of host control. Expression of several m6A RNA-methylation regulators varies with disease severity. Fatal cases show increased expression of the demethylase ALKBH5 and the m6A reader YTHDF2, while the demethylase FTO is elevated in severe disease [307]. These findings suggest that altered m6A-mediated regulation of RNA stability and translation may influence antiviral signaling and viral replication [307].

Genetic variation influencing chemokine signaling may further modulate inflammatory responses during CCHFV infection. Monocyte chemoattractant protein-1 (MCP-1/CCL2), which recruits monocytes through CCR2 signaling, has been implicated in disease severity. In a study of 128 patients with confirmed CCHF, the MCP-1  polymorphism (-2518 A/G) was associated with clinical outcome: the AA genotype and A allele were correlated with severe disease, whereas the AG genotype was associated with milder disease [308].

### Endothelial and vascular genetic determinants

Genetic determinants affecting endothelial function and vascular signaling may further contribute to CCHF pathogenesis. Variations in VEGF influence endothelial activation, vascular permeability, and angiogenesis.

In a study of 130 individuals including 60 CCHF patients, VEGF (−460 C/T) and ID (−2549 18 bp) promoter polymorphisms differed significantly between patients and controls, with TT and TC genotypes more common among patients [309]. Similarly, VEGF 936 C /T polymorphisms were associated with increased susceptibility, with individuals carrying the C/C genotype or C allele showing higher risk [310]. In contrast, polymorphisms in the VEGF receptor KDR (−604 *T* / C) were not associated with disease susceptibility.

Endothelial function is also influenced by nitric oxide (NO) produced by endothelial nitric oxide synthase (eNOS). In a cohort of 54 CCHF patients, the eNOS T786C polymorphism was significantly more frequent in patients than controls, whereas the 4a/4b variant showed no association with disease [311].

## Key knowledge gaps in CCHFV pathogenesis

Although substantial progress has been made in understanding CCHFV, key aspects of viral maintenance, host infection, and disease progression remain poorly defined. Important gaps span the enzootic cycle in ticks and vertebrate hosts as well as the mechanisms that drive severe disease in humans. Addressing these questions will require integrated investigation across vector biology, vertebrate host responses, and human immunopathogenesis.

### Missing links in tick-mediated viral maintenance and amplification

Long-term maintenance of CCHFV in tick populations is fundamental to the virus’s ecology, yet the mechanisms that enable persistent infection without overt pathology remain poorly defined. *Hyalomma* ticks function as both reservoirs and vectors; however, the molecular and cellular processes that support lifelong viral maintenance while preserving tick fitness have yet to be fully elucidated. Experimental observations suggest that CCHFV infection does not measurably impair feeding success or egg production in *Hyalomma* ticks. However, given the long lifespan of hard ticks, subtler effects on longevity, questing behavior, offspring fitness, or long-term vector competence remain plausible but have not been systematically examined.

At the mechanistic level, important gaps remain in understanding how CCHFV interacts with tick immune defenses. Ticks possess relatively simple but functional innate immune pathways, including Toll, JAK/STAT, and RNA interference, that can be modulated by infection [312]. Studies of other tick-borne pathogens demonstrate that microbes can alter Toll signaling and MyD88 expression to promote persistence within the vector [313]. The selective pressures associated with long-term persistence in ticks have been shown to drive significant viral genetic diversification and the emergence of consensus-level mutations [40]. Characterizing how CCHFV interacts with and navigates tick immune pathways may provide important insights into the mechanisms that shape viral infectivity, adaptation, and genome evolution within the tick host. Tick hemocytes are pleiotropic immune cells involved in pathogen recognition, phagocytosis, and immune signaling, yet their responses to viral infection, particularly CCHFV infection, remain largely unexplored. Similarly, infectious organisms can induce changes in tick miRNAs and cellular metabolism, suggesting that pathogens may reprogram vector immunity and physiology in ways that influence vector competence; however, mechanistic studies specific to CCHFV have not been conducted [64,312,314,315]. As a result, it remains unclear whether CCHFV establishes a stable, endosymbiont-like relationship within tick tissues or persists through transient cycles of replication and immune tolerance.

Beyond intrinsic immune interactions, ecological and biological modifiers of infection remain poorly defined. These include the effects of microbiome composition and co-infection on viral replication and transmission, as well as potential developmental stage-specific differences in susceptibility. Tick saliva contains numerous immunomodulatory molecules that facilitate feeding and pathogen transmission, yet the extent to which these factors influence CCHFV replication within the tick or shape early host infection through saliva-assisted transmission remains unclear [316].

Key unresolved questions in tick infection include:

- What intrinsic features of *Hyalomma* ticks permit efficient viral acquisition and lifelong maintenance without tissue damage, and why are other arthropods less permissive?
- Do tick immune pathways and pleiotropic hemocytes promote controlled persistence rather than clearance, and how does CCHFV interact with these cells to avoid pathogenic inflammation?
- Do microbiome composition, co-infection, or developmental stage shape viral replication and tolerance?
- Does infection induce subtle metabolic or cellular stress that affects tick fitness or behavior while preserving vector competence?

Understanding how CCHFV persists in ticks without causing disease may provide a broader model for viral tolerance and identify mechanisms that, when dysregulated in vertebrates, contribute to pathology.

### Missing links in vertebrate host infection, immunity, and virulence

In contrast to the severe disease observed in humans, infection of vertebrate reservoir hosts is typically asymptomatic despite the presence of viremia sufficient to infect feeding ticks. Domestic and wild ungulates therefore play a central role in enzootic maintenance of CCHFV, yet the mechanisms that allow viral replication while preventing immunopathology remain poorly understood.

A major gap is the limited characterization of viral tissue tropism in reservoir hosts. In humans and nonhuman primates, monocytes and macrophages are recognized targets of infection. Whether similar cell populations support productive infection in ungulates remains unclear, as does whether viral replication is spatially or temporally restricted. Systematic evaluation of viral distribution across tissues, including lymphoid organs, liver, spleen, endothelium, bone marrow, and skin, has not been performed in most ungulate models. Determining whether infection is rapidly contained, compartmentalized, or restricted to specific cell populations could help explain the absence of overt pathology.

Important gaps also remain in understanding host immune responses. Ungulates typically develop brief viremia without clinical disease, suggesting effective early control or tolerance of infection. However, the mechanisms underlying this outcome remain undefined. Potential explanations include differences in mononuclear cell permissiveness, IFN signaling, cytokine regulation, or susceptibility to viral immune antagonism. Comparative studies across species are needed to determine whether reservoir hosts mount qualitatively distinct innate responses or instead limit tissue damage through tighter regulation of inflammation.

Additional uncertainties involve biological modifiers of infection. Factors such as age, pregnancy, immune status, co-infection, and microbiome composition may influence viral dynamics and transmission potential but remain poorly studied. Similarly, the extent to which endothelial activation occurs in reservoir hosts is unknown, despite its central role in vascular pathology during severe human disease.

Key unresolved questions in host infection include:

- Do the cellular receptors and entry pathways utilized by CCHFV differ between ungulate hosts, humans, and ticks, as suggested by *in vitro* studies?
- Which cell types support viral replication in reservoir species, and how do viral replication kinetics differ from those observed in humans?
- Do ungulates rapidly clear infection or do they tolerate limited viral replication through tightly regulated inflammatory responses? How do these mechanisms differ from those in humans?
- Is endothelial activation limited in these species, thereby minimizing vascular dysfunction and pathology?
- Does CCHFV persist transiently within specific tissues long enough to facilitate transmission to feeding ticks, or can infection recrudesce under certain conditions?
- How do host factors, including age, pregnancy status, immune status, co-infection, and microbiome composition, influence viral dynamics and transmission potential?

Integrating vertebrate immunology with vector biology will be essential to fully understand enzootic maintenance and how asymptomatic infection contributes to transmission. These comparisons may also identify host pathways that protect against immunopathology in humans.

### Knowledge gaps in the mechanisms of severe human disease

Severe CCHFV infection is characterized by dysregulated immune responses, vascular injury, and multiorgan involvement, yet the mechanisms that drive progression from mild infection to severe disease remain poorly defined. Much of the available evidence derives from animal models, observational clinical cohorts, or cross-sectional transcriptomic studies, which limits the ability to identify causal drivers of pathogenesis.

A major limitation in the current literature is the lack of functionally validated immunologic and transcriptional studies in human infection. IFN responses, inflammatory signatures, and dysregulated immune pathways have been described in multiple studies, yet these observations are rarely tested in mechanistic systems using primary human cells or validated through longitudinal patient sampling. Consequently, it remains difficult to distinguish pathways that actively drive pathology from those that reflect downstream responses to viral replication and systemic inflammation.

Another major gap concerns the temporal dynamics of disease progression. Most human studies rely on samples collected at a single time point, often at hospital admission, making it difficult to reconstruct the sequence of viral replication, immune activation, and tissue injury. Longitudinal datasets spanning asymptomatic, mild, severe, and fatal cases would help determine whether severe disease reflects early failure of antiviral control, delayed IFN responses, excessive inflammatory amplification, or dysregulated tissue repair. Understanding how immune signatures evolve over time, particularly in patients who deteriorate after an initially mild presentation, may reveal critical transition points in pathogenesis.

Host genetic contributions to disease severity also remain incompletely defined. Associations between specific polymorphisms and severe outcomes have been reported, but most studies have been geographically limited and conducted in relatively small cohorts. Larger multi-regional analyses are needed to determine whether these associations are broadly applicable and to clarify how host and viral genetic diversity interact to shape clinical outcomes.

Key unresolved questions in human disease include:

- What host factors distinguish asymptomatic or mild infection from severe disease?
- How do the infection dynamics and functional responses of monocytes and macrophages differ between mild and severe disease?
- How do viral load, type I IFN responses, and inflammatory mediators interact over time to shape disease progression and clinical outcome?

Progress will depend on coordinated clinical and mechanistic studies integrating longitudinal patient sampling, functional immunology, and viral genomics. These approaches may help define pathways that drive immunopathology and identify host mechanisms that limit disease severity.

## Conclusion

Current evidence defines the broad ecological and pathogenic framework of CCHFV while highlighting critical mechanistic gaps. The virus is maintained in enzootic cycles involving ixodid ticks and a diverse range of vertebrate hosts that develop transient viremia but rarely exhibit overt disease. In contrast, human infection can progress to a dysregulated inflammatory syndrome characterized by vascular injury, coagulopathy, multiorgan dysfunction, and, in severe cases, death.

Despite this foundational understanding, the mechanisms that distinguish tolerant infection in ticks and vertebrate hosts from pathogenic infection in humans remain incompletely defined. Key uncertainties include how CCHFV persists in reservoir ticks without compromising fitness, how reservoir hosts control viral replication without developing disease, and why similar host-virus interactions in humans can lead to immune dysregulation and systemic pathology.

Addressing these questions will require integrated investigations spanning vector biology, reservoir host infection, and human immunopathogenesis. Comparative studies examining how infection is contained or tolerated across species may reveal host pathways that limit pathology in natural reservoirs and identify mechanisms driving severe disease in humans. These insights will be critical for defining the determinants of CCHFV virulence and for informing strategies to prevent and manage severe disease.

## Supplementary Material

Supplemental Table 1_Clinpath data.xlsx

Supplemental Table 3_CS_surv_vs_nonsurv.xlsx

Supplemental Table 2_CS_Mod_vs_severe_Resub.xlsx

## Data Availability

The data supporting this review are derived from previously published studies and publicly available sources, all of which are cited within the article. No new datasets were generated or analyzed for this study.
